# A Linear Superposition Model of Envelope and Frequency Following Responses May Help Identify Generators Based on Latency

**DOI:** 10.1162/nol_a_00072

**Published:** 2022-07-19

**Authors:** Tobias Teichert, G. Nike Gnanateja, Srivatsun Sadagopan, Bharath Chandrasekaran

**Affiliations:** 1 Department of Psychiatry, University of Pittsburgh, Pittsburgh, PA, USA; 2 Department of Bioengineering, University of Pittsburgh, Pittsburgh, PA, USA; 3 Center for Neuroscience, University of Pittsburgh, Pittsburgh, PA, USA; 4 Department of Communication Sciences and Disorders, University of Pittsburgh, Pittsburgh, PA, USA; 5 Department of Neurobiology, University of Pittsburgh, Pittsburgh, PA, USA

**Keywords:** envelope following responses, frequency following responses, temporal fine structure, macaque monkey, EEG, deconvolution

## Abstract

Envelope and frequency-following responses (FFR_ENV_ and FFR_TFS_) are scalp-recorded electrophysiological potentials that closely follow the periodicity of complex sounds such as speech. These signals have been established as important biomarkers in speech and learning disorders. However, despite important advances, it has remained challenging to map altered FFR_ENV_ and FFR_TFS_ to altered processing in specific brain regions. Here we explore the utility of a deconvolution approach based on the assumption that FFR_ENV_ and FFR_TFS_ reflect the linear superposition of responses that are triggered by the glottal pulse in each cycle of the fundamental frequency (F0 responses). We tested the deconvolution method by applying it to FFR_ENV_ and FFR_TFS_ of rhesus monkeys to human speech and click trains with time-varying pitch patterns. Our analyses show that F0_ENV_ responses could be measured with high signal-to-noise ratio and featured several spectro-temporally and topographically distinct components that likely reflect the activation of brainstem (<5 ms; 200–1000 Hz), midbrain (5–15 ms; 100–250 Hz), and cortex (15–35 ms; ~90 Hz). In contrast, F0_TFS_ responses contained only one spectro-temporal component that likely reflected activity in the midbrain. In summary, our results support the notion that the latency of F0 components map meaningfully onto successive processing stages. This opens the possibility that pathologically altered FFR_ENV_ or FFR_TFS_ may be linked to altered F0_ENV_ or F0_TFS_ and from there to specific processing stages and ultimately spatially targeted interventions.

## BACKGROUND

Envelope and frequency-following responses (FFR_ENV_ and FFR_TFS_) are scalp-recorded electrophysiological potentials that closely follow the periodicity of complex sounds such as speech (Aiken & Picton, 2008; Chandrasekaran & Kraus, 2010; Skoe & Kraus, 2010). Initially thought to reflect activity arising mostly from the cochlear nucleus and inferior colliculus (Chandrasekaran & Kraus, 2010), current thinking assumes multiple sources distributed across brainstem, midbrain, and cortex (Coffey et al., 2019). Over the past two decades, altered FFR_ENV_ and FFR_TFS_ have been established as an important biomarker in speech and learning disorders (Anderson et al., 2010; Banai et al., 2005, 2009; Chandrasekaran et al., 2009; Cunningham et al., 2001; Hornickel et al., 2012; Hornickel & Kraus, 2013; Russo et al., 2009). Given the emerging view of FFR_ENV_ and FFR_TFS_ as signals arising from widely distributed sources, there are many different potential anatomical substrates for pathologically altered responses. Understanding the anatomical substrate of altered FFR_ENV_ and FFR_TFS_ is a critical first step in the process of understanding and ultimately ameliorating the deficits with spatially targeted interventions. However, despite important advances, it has remained challenging to map altered FFR_ENV_ and FFR_TFS_ features to altered processing in specific brain regions. As a result, the potential of FFR_ENV_ and FFR_TFS_ to reveal spatially specific insights into the function of different auditory processing stages has not been fully unlocked.

For “classical” auditory evoked onset responses, important information about the neural origin can be gleaned from their latency and topography. Depending on their latency, neural responses have been coarsely attributed to auditory brainstem (<10 ms), midbrain (10–50 ms), or cortex (>50 ms) (Alain & Winkler, 2012). Topography, i.e., the spatial distribution of electric or magnetic fields across the scalp, can then be analyzed using source modeling approaches to further narrow down the exact spatial location of the underlying neural generators. Recent work has shown that source modeling can also be leveraged to better understand the neural generators of the FFR (Bidelman, 2015; Coffey et al., 2016; Gerken et al., 1975; Gorina-Careta et al., 2021). However, because of its dependence on high channel-count electroencephalograph (EEG) and/or magnetoencephalograph (MEG) recordings, source modeling is often not feasible for clinical FFR_ENV_ and FFR_TFS_ data which is typically recorded with a 3-electrode montage.

**Figure 1.**
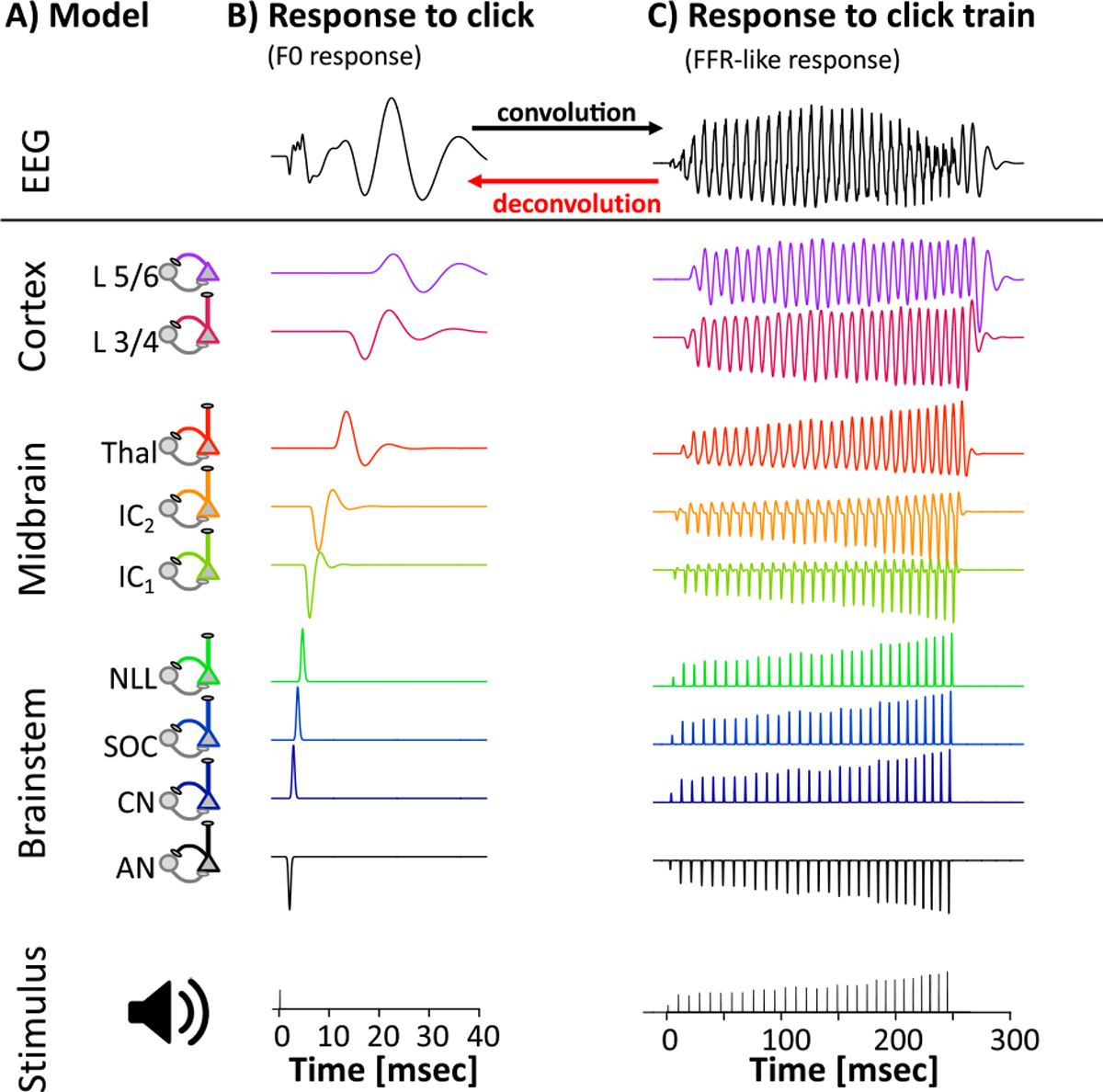
Linear superposition hypothesis of the FFR. (A) Schematic of a hypothetical feedforward model with nodes in brainstem, midbrain, and cortex (AN: auditory nerve, CN: cochlear nucleus, SOC: superior olivary complex, NLL: nucleus of lateral lemniscus, IC_1,2_ inferior colliculus, Thal: thalamus, L3/4, L5/6: layer 3/4 and 5/6 of primary auditory cortex). (B) Response of each node to a single click-like event (F0 response). Hypothetical EEG response arises as the sum of activity over all nodes. (C) Because the model is linear, the response to several click-like events in close temporal proximity (FFR-like response) is identical to the sum of the same events presented in isolation (convolution). In theory, the F0 response can be recovered from the FFR-like response using deconvolution.

An alternative approach can be derived from the hypothesis that FFR_ENV_ and/or FFR_TFS_ reflect the linear superposition of responses to each glottal pulse (F0 response) that sequentially activates processing stages in brainstem, midbrain, and cortex (Figure 1) (Bidelman, 2015; Dau, 2003; Gerken et al., 1975; Janssen et al., 1991). Despite its theoretical relevance, the superposition hypothesis has not been subject to much empirical scrutiny (Bidelman, 2015). If the superposition hypothesis is accurate, FFR_ENV_ and/or FFR_TFS_ would arise as the convolution of the F0 response with a series of impulses, mathematically described as Dirac pulses, whose time and amplitude reflect the onset and intensity of each glottal pulse, or more generally, each F0 cycle. Furthermore, it should be possible to compute the underlying F0 responses by deconvolution. *Deconvolution* approaches have successfully been used in a wide range of neuroscientific applications (Aquino et al., 2014; Teichert & Ferrera, 2015), including the closely related 40 Hz auditory steady state response (Bohórquez & Özdamar, 2008) and continuous speech (Maddox & Lee, 2018; Polonenko & Maddox, 2021). To date, however, deconvolution has never been used to recover the F0 response underlying FFR_ENV_ or FFR_TFS_ to stimuli with time-varying pitch. Thus, it is unknown how well a linear superposition model can account for the considerable spectro-temporal complexity of FFR_ENV_ and FFR_TFS_, and how much of their variance it can capture. If the F0 responses indeed account for a substantial portion of FFR_ENV_ and/or FFR_TFS_, they may provide useful information about the functional integrity of the different generators underlying FFR_ENV_ and/or FFR_TFS_.

Based on results from the 40 Hz steady state response and continuous speech (Bohórquez & Özdamar, 2008; Maddox & Lee, 2018; Polonenko & Maddox, 2021), we hypothesized that the F0_ENV_ responses can explain a large portion of the FFR_ENV_. It is less clear if the same would be true for the FFR_TFS_. If successful at explaining much of the variance, the F0_ENV_ and F0_TFS_ may help link altered FFR_ENV_ and FFR_TFS_ to altered function in specific auditory processing stages. As a first step in that direction, we addressed three main questions: (i) What percentage of the variance of FFR_ENV_ and FFR_TFS_ can be explained by the linear superposition of F0 responses? (ii) How reliably can F0 responses be estimated? (iii) Is there any evidence that the latencies of F0 responses can be linked to anatomically distinct processing stages?

We decided to perform our experiments in macaque monkeys for three reasons: First, the monkey is a well-established model for the human auditory system in general because their auditory system shares important functional (Bigelow & Poremba, 2014; Fishman & Steinschneider, 2012; Gil-da-Costa et al., 2013; Javitt et al., 2000; Steinschneider et al., 1992) and structural (Chaplin et al., 2013; Sweet et al., 2005) similarities with humans. Second, macaques are known to exhibit human-like FFR_ENV_ (Ayala et al., 2017; Brugge et al., 2009; Fishman et al., 2013; Gnanateja et al., 2021; Steinschneider et al., 1998, 2003). Third, this species will ultimately allow us to use invasive recordings to confirm the predictions of the deconvolution method by directly measuring FFR_ENV_ and FFR_TFS_ along different stages of the auditory pathway.

## METHODS

### Subjects

Data reported here was collected from two adult male macaque monkeys (*Macaca mulatta*). All experiments were performed in accordance with the guidelines set by the U.S. Department of Health and Human Services (National Institutes of Health) for the care and use of laboratory animals. All methods were approved by the Institutional Animal Care and Use Committee at the University of Pittsburgh. The animals had previously been exposed to pure tone and click-stimuli in passive and active listening paradigms.

### Stimuli

**Figure 2.**
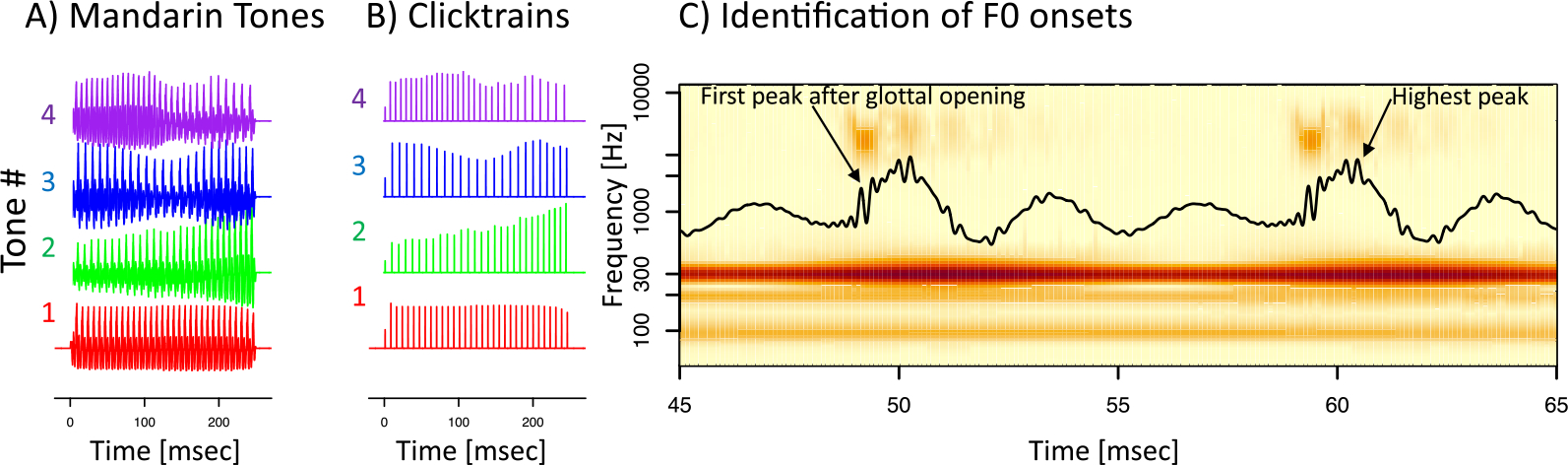
Stimuli. (A) The four synthetic Mandarin tones in the time domain. (B) The corresponding click train stimuli. (C) A snippet containing two F0 cycles of a Mandarin tone stimulus in the time (black line) and time-frequency domain (color). Timing of the clicks in the click train stimuli matched the time of the highest pressure peak (second F0 cycle). We subsequently defined the onset of an F0 cycle as the first positive pressure peak that coincides with the first of several peaks of power in the third formant that follows the opening of the glottis (first F0 cycle).

Two types of stimuli were used: (a) synthesized Mandarin tones (Figure 2A) and (b) click train versions thereof (Figure 2B). *Mandarin tones*: The synthesized Mandarin tones used the vowel /yi/ in the context of four distinct F0 patterns: T1 (high-level, F0 = 129 Hz), T2 (low-rising, F0 ranging from 109 to 133 Hz), T3 (low-dipping, F0 ranging from 89 to 111 Hz), and T4 (high-falling, F0 ranging from 140 to 92 Hz). Mandarin tones were synthesized based on the F0 patterns derived from natural male speech production (Xie et al., 2017). All stimuli had a sampling rate 96000 Hz and were 250 ms in duration and were presented at 78 dB SPL. The stimuli were presented in both condensation and rarefaction polarities. By computing either the sum or the difference of the two polarities, it was possible to highlight the neural responses to either the temporal periodicity envelope (FFR_ENV_) or the temporal fine structure (FFR_TFS_) (Krizman & Kraus, 2019).

The stimuli were presented in a randomized manner, with randomly selected inter-stimulus intervals between 300 and 500 ms. In each 40 min long recording session, we presented 500 repetitions of each tone and polarity for a total of 4,000 sweeps. *Click train stimuli*: From each of the four synthesized Mandarin tone stimuli, we prepared a click train version that consisted of trains of 0.1 ms long monophasic impulses. Timing and amplitude of the clicks in the click trains matched the timing and amplitude of the F0 cycles of the Mandarin tone stimuli. The timing of the F0 cycles was operationalized as the time of the peak pressure (Figure 2C, second F0 cycle); the intensity was operationalized as twice the absolute amplitude of the peak activity to account for the fact that speech sounds are modulated bi-directionally.

The rationale for using the Mandarin tone sets was twofold. First, we were interested in using a stimulus set that had already been used to study FFR_ENV_ and FFR_TFS_ in numerous basic and clinical studies (Chandrasekaran et al., 2014; Lau et al., 2021; Xie et al., 2018). If successful, the deconvolution technique may be able to extract further information from these existing data sets. Second, we were interested in a stimulus set with a wide range of fundamental frequencies, because otherwise the solution to the deconvolution is not unique. We introduced the click-train stimuli to create a scenario that would be particularly amenable to the superposition hypothesis and thus to our deconvolution-based analytic approach.

### Experimental Setup

All experiments were performed in a small (4′ × 4′ × 8′) sound-attenuating and electrically insulated recording booth (Eckel Noise Control Technology). The animal was positioned and head-fixed in a custom-made primate chair (Scientific Design). Neural signals were recorded at a sampling rate of 30 kHz with a 256-channel digital amplifier system (RHD2000, Intan).

Experimental control was handled by a Windows PC running an in-house modified version of the Matlab (https://www.mathworks.com/) software package *monkeylogic*. Sound files were generated prior to the experiments and presented by a subroutine of the Matlab package *Psychtoolbox*. The sound files were presented using the right audio channel of a high-definition stereo PCI sound card (M-192 from M-Audiophile) operating at a sampling rate of 96 kHz and 24-bit resolution. The analog audio signal was then amplified by a 300-watt amplifier (QSC GX3). The amplified electric signals were converted to sound waves using a single element 4-inch full-range driver (Tang Band W4–1879) located 20 cm in front of the animals. Over the relevant range of presented frequencies the sound pressure level of the speaker varied ±7.5 dB SPL.

To determine sound onset with high accuracy, a trigger signal was routed through the unused left audio channel of the sound card directly to one of the analog inputs of the recording system. Thus, sound onset could be determined at a level of accuracy that was limited only by the sampling frequency of the recording device (30 kHz: corresponding to 33 μs).

### Cranial EEG

EEG activity was recorded from 33 EEG electrodes that were chronically implanted in 1 mm deep non-penetrating holes in the cranium (Purcell et al., 2013; Teichert, 2016; Woodman et al., 2007). Electrodes were positioned across the entire accessible part of the cranium at positions approximately homolog to the international 10–20 system in the human (Li & Teichert, 2020). More details of the EEG recording setup have been provided in earlier work (Teichert, 2016; Teichert et al., 2016). Data were recorded with an Intan RHD 2000 digital amplifier. The midline electrode immediately anterior to Oz served as the recording reference and ground electrode. Data were referenced offline to the Oz electrode. In one animal, all electrodes were functional, allowing us to perform the deconvolution for all electrodes and thus visualize topographies of the F0 responses. In the second animal only a subset of the electrodes was functional, thus preventing topographical analyses.

### Pre-Processing

The raw data were band-pass filtered using a second-order zero-phase shift Butterworth filter with cutoff frequencies of 60 and 2000 Hz. Time-locked epochs were extracted and down-sampled to a rate of 10 kHz. Epochs that exceeded an artifact-rejection criterion based on the distribution of peak-to-peak amplitudes for each individual channel were excluded from further analyses for that channel. If an epoch exceeded the relative amplitude criterion in two or more channels, it was rejected for all channels. This relative amplitude criterion allowed us to process a range of channels with different noise levels simultaneously, i.e., using the same (relative) criterion. The valid epochs were averaged separately for the four tones to obtain a total of four waveforms. In addition, the valid epochs were also averaged separately for all tones and polarity to obtain eight waveforms.

### Deconvolution Approach

#### Click trains

The starting points for the click train deconvolution approach were click onset times and their amplitudes. The amplitudes were further normalized to an average value of 1 across all 4 click trains. The onset times were then shifted in steps of 0.1 ms (i.e., the sampling rate of the data) between 0 and 79.0 ms, for a total of 800 regressors. We then fit a linear model to the FFR_ENV_ and FFR_TFS_ using all 800 regressors. To that aim, FFR_ENV_ or FFR_TFS_, respectively, from all stimuli and the corresponding regressors were concatenated into a single time series padded with NaN (Not a Number) values between them to avoid cross talk between the end of one stimulus and the beginning of the next. The FFR_ENV_ or FFR_TFS_ kernel was then defined as the weights of the 800 regressors. The deconvolution approach thus identified the kernel that best explained the observed FFR_ENV_ or FFR_TFS_ as the linear sum of overlapping responses to each individual click in the click train. The time axis of the kernel thus corresponded to time after click onset. Similarly, the latency of specific components of the kernel were measured in time after click onset. Note that the FFR_ENV_ or FFR_TFS_ to all four stimuli were explained by a single 80 ms long kernel. The deconvolution approach was implemented in the statistical software R, using an in-house written deconvolution package (deconvolvR).

#### Mandarin tones

An almost identical procedure was used to create the predictors for the tone FFR_ENV_ and FFR_TFS_. However, to create the click trains, we placed individual clicks at the time of the peak pressure of each F0 cycle (Figure 1C, second F0 cycle). This choice may have been suboptimal, as peak pressure does not coincide with the timing of the actual glottal pulse. We thus identified an approach and operationalized the onset of each F0 cycle as the first positive pressure peak that coincided with a peak of power in the third harmonic (Figure 1C, first F0 cycle). The two different approaches yielded highly similar timing, but the estimated F0 onsets preceded the time of peak pressure very reliably by 1.01 ms. Tone FFR_ENV_ kernels were estimated from both types of predictors based on the timing of the peak pressure and glottal pulse. Both yielded almost identical results. However, the FFR_ENV_ kernels from the peak pressure were delayed by approximately 1 ms, and they explained a somewhat lower amount of variance. Furthermore, the timing of the tone kernel based on the glottal pulse matched the timing of the click kernel much better than the tone kernel based on peak pressure. Following the theoretical arguments and the empirical support, we report the tone FFR_ENV_ and FFR_TFS_ kernels using the glottal onset time rather than the time of peak pressure.

### Nonlinear–Linear Model

We also developed a nonlinear–linear model to account for a very specific limitation of the linear deconvolution model. The first nonlinear stage of the model accounts for short-term adaptation in the early auditory system. The short-term adaptation stage uses two parameters, tau and U, to estimate how quickly and how strongly early stages of the auditory system adapt to the repeated F0 onsets (Teichert et al., 2016). The parameters U and tau were estimated using a gradient descent approach. The output of the nonlinear stage corresponds to a series of Dirac pulses whose amplitude reflects both the amplitude of the F0 cycle, and the degree of adaptation accrued by responses to previous F0 cycles.

In addition to the nonlinear stage, we also modified the linear stage to include a stimulus onset regressor. This allows for the possibility that the very first F0 onset triggers a stimulus onset response that is qualitatively distinct from the remaining F0 responses. To keep the number of regressors similar, we reduced the lag from 80 ms (800 regressors) to 45 ms for both types of response (2 × 450 = 900 regressors). As before, the model was fit to the training set, and model fit was evaluated in the testing set.

### Quantification of Model Fit

The primary variable used to quantify the quality of the model fit was percentage variance explained. Percentage variance explained is typically calculated as 100 * (TMS − RMS)/TMS. Here RMS stands for the mean of the squares of the residuals, and TMS for the mean of the squares of the total signal, i.e., including variance pertaining to the actual FFR as well as measurement noise. Since no model can be expected to account for measurement noise, this traditional metric cannot reach 100% unless there is no measurement noise. The limit of percentage variance a model can explain is given by 100 − 100/signal-to-noise ratio. As a result, the metric is only comparable for data sets with similar signal-to-noise ratio. Because some of our recording sessions have a range of different signal-to-noise ratios, we decided to use an alternative metric that adjusts for different signal-to-noise ratios. This metric sets out to quantify how much of the “explainable” variance, i.e., the portion of the variance that exceeds the variance of the baseline, can be explained by the model: 100 * (TMS − RMS)/(TMS − BMS). In this context, BMS stands for the mean of the squares of the signal on the baseline, defined as the 50 ms period before stimulus onset, and the period from 320 to 390 ms after stimulus onset, i.e., 70 to 140 ms after stimulus offset. We had found the variance on the post-stimulus baseline to be systematically smaller than on the pre-stimulus baseline. Hence the decision to use the average of both periods.

Unless mentioned otherwise, we will refer to this signal-to-noise ratio-corrected measure of percentage variance explained throughout the article. Percentage variance explained was calculated across the entire simulation period (0 to 280 ms after stimulus onset), as well as the sustained period which excluded both on- and offset responses (50 to 250 ms). Note that in all cases, the kernel was estimated by fitting it to the entire temporal duration of the data. Consequently, any difference in percentage variance explained is not caused by requiring the model to fit a simpler subset of the data, but rather depends on how well the same underlying model accounts for the data in different epochs.

Furthermore, we performed a wavelet decomposition of the signal as well as the residuals and evaluated percentage variance explained in three different frequency bands, the frequency range of the fundamental frequency F0 (70–170 Hz), the frequency range of the first formant F1 (180–300 Hz), and the frequency range of harmonics beyond the first formant Fx (400–1200 Hz). To account for the temporal smearing of the wavelet decomposition, the time ranges of all periods were shrunk by 20 ms on each side.

#### Data split control

To prevent overfitting caused by determining the kernel and the percentage variance explained from the same data set, we randomly split the data of each recording session into two equally sized subsets. The first subset of data (training set) was used to estimate the kernel. This kernel was then used to determine percentage variance explained of the second subset (testing set). In the context of the work presented here, the approach was only used for the data averaged across all sessions.

#### Cross-day control

At the single session level, we used a different approach to prevent overfitting. Specifically, to explain FFR_ENV_ from one recording session we only used kernels extracted from different recording sessions. The data fit metric for the session in question, e.g., percentage variance explained, was then defined as the average of that metric using kernels from all other sessions.

#### Shuffle control

To control for the large number of predictors in the linear model (80 [ms] × 10 [samples per ms] = 800) we included a shuffle-control. The shuffle control used the same averaged data and the same predictors. However, the timing of the Dirac pulses was shuffled such that the timing and amplitude designed to match the F0 onsets for tone 2 were used to predict data for tone 1, the timing and amplitude designed for tone 3 were used for tone 2, and so on. This approach was used for data averaged across all recording sessions as well as for data of individual recording sessions.

### Data Quality and Rejection of Recording Sessions

For the click train stimuli we recorded a total of 31 EEG sessions (animal B: 17, animal J: 14). For the Mandarin tone stimuli we recorded a total of 20 EEG sessions (animal B: 2, animal J: 18). Sessions were included in the analyses if the noise of the averaged FFRs on the baseline was below 0.008 uV^2^. Data quality for animal J was variable between sessions, and approximately half of the sessions did not meet the criterion (animal J, click train stimuli: 8/14 sessions; tone: 9/18 sessions). Data quality for animal B was consistently high. Only one of the click train sessions needed to be excluded because of noise. In addition, we excluded one of the click train sessions because the signal amplitude was less than half of the other sessions, a clear outlier given the tight distribution of values for the other sessions. In summary, we used 2/2 tone sessions and 15/17 click train sessions for animal B.

Noise amplitude on the excluded sessions were distributed bimodally: a small fraction of recording sessions with an increase of well over tenfold, and a larger fraction with an increase below twofold. Including the sessions with less than a twofold increase did not change the main conclusions. However, it did increase variability of the results between sessions and decrease the percentage variance explained by a relatively modest amount. The key takeaway from including the noisier sessions is not very unexpected: If data quality is lower, less variance can be explained.

## RESULTS

Electrophysiological responses were recorded in response to two types of stimuli: (i) four synthetic Mandarin tones using the syllable /yi/ and (ii) click train versions of these Mandarin tone stimuli. Click train stimuli were created by converting the four Mandarin tone stimuli into series of monophasic clicks whose timing and amplitude matched the estimated time of onset of each F0 cycle (Figure 2A, see Methods for details). We report data from a total of 23 EEG recording sessions using the click train stimuli (15 sessions animal B; 8 sessions animal J) and 11 sessions using the Mandarin tone stimuli (2 sessions animal B; 9 sessions animal J). Each session lasted 40 min and contained a total of 4,000 stimuli, 500 from each type and polarity.

By computing either the sum or the difference of responses to the two polarities, the data can highlight either the neural responses to individual cycles of carrier frequencies below a physiological threshold (FFR_TFS_), or neural responses to periodic envelope modulations (FFR_ENV_). Our results will initially focus on data averaged across both polarities, and thus FFR_ENV_. The second half of the Results section will focus on difference between polarities, and thus the FFR_TFS_.

### Tone and Click Train FFR_ENV_

**Figure 3.**
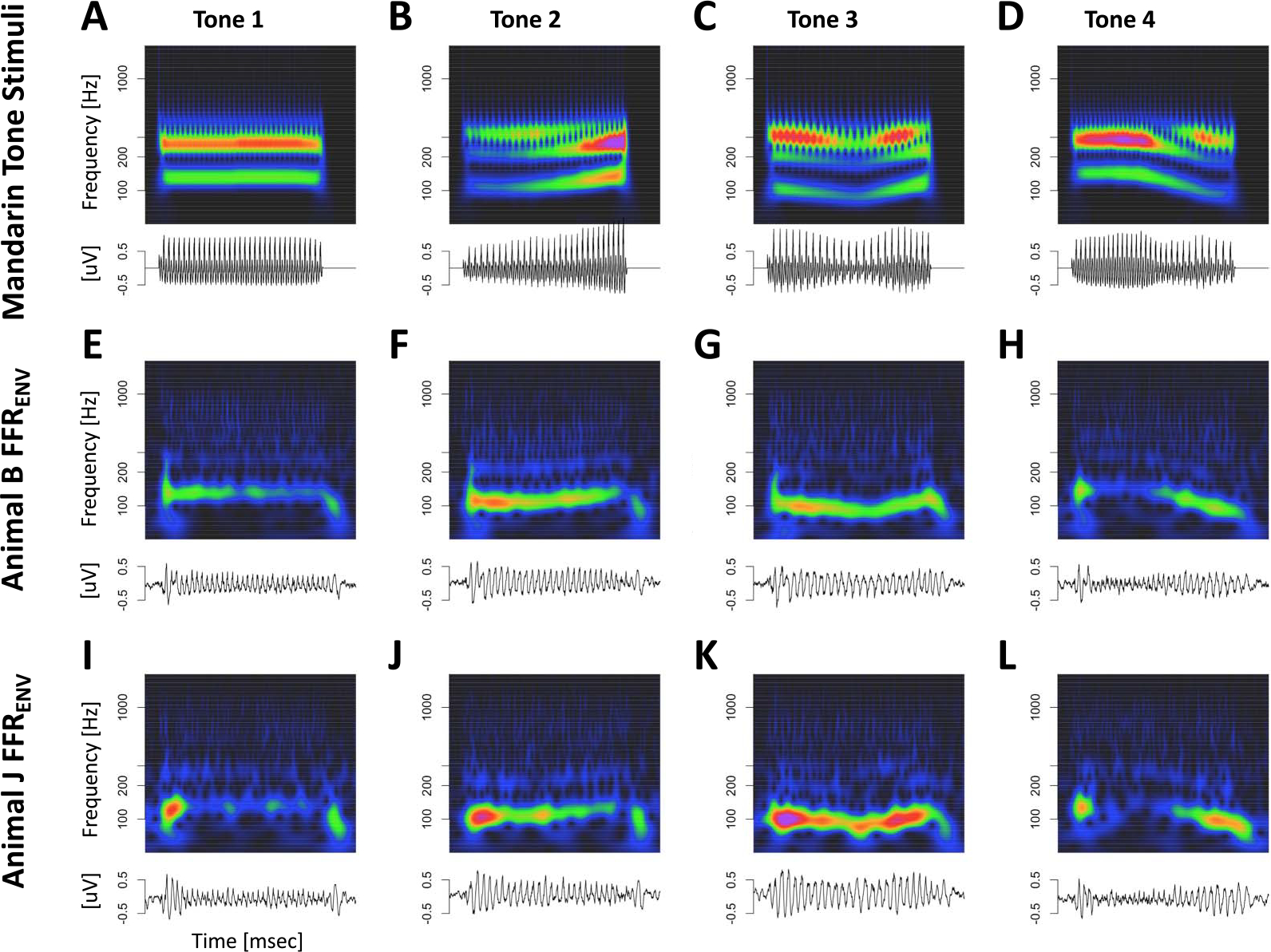
Mandarin Tone FFR_ENV_. Representation of Mandarin tone stimuli and the corresponding FFR_ENV_ in the time and time-frequency domain. (A–D) Stimuli. (E–F) Monkey B FFR_ENV_. (I–L) Monkey J FFR_ENV_.

**Figure 4.**
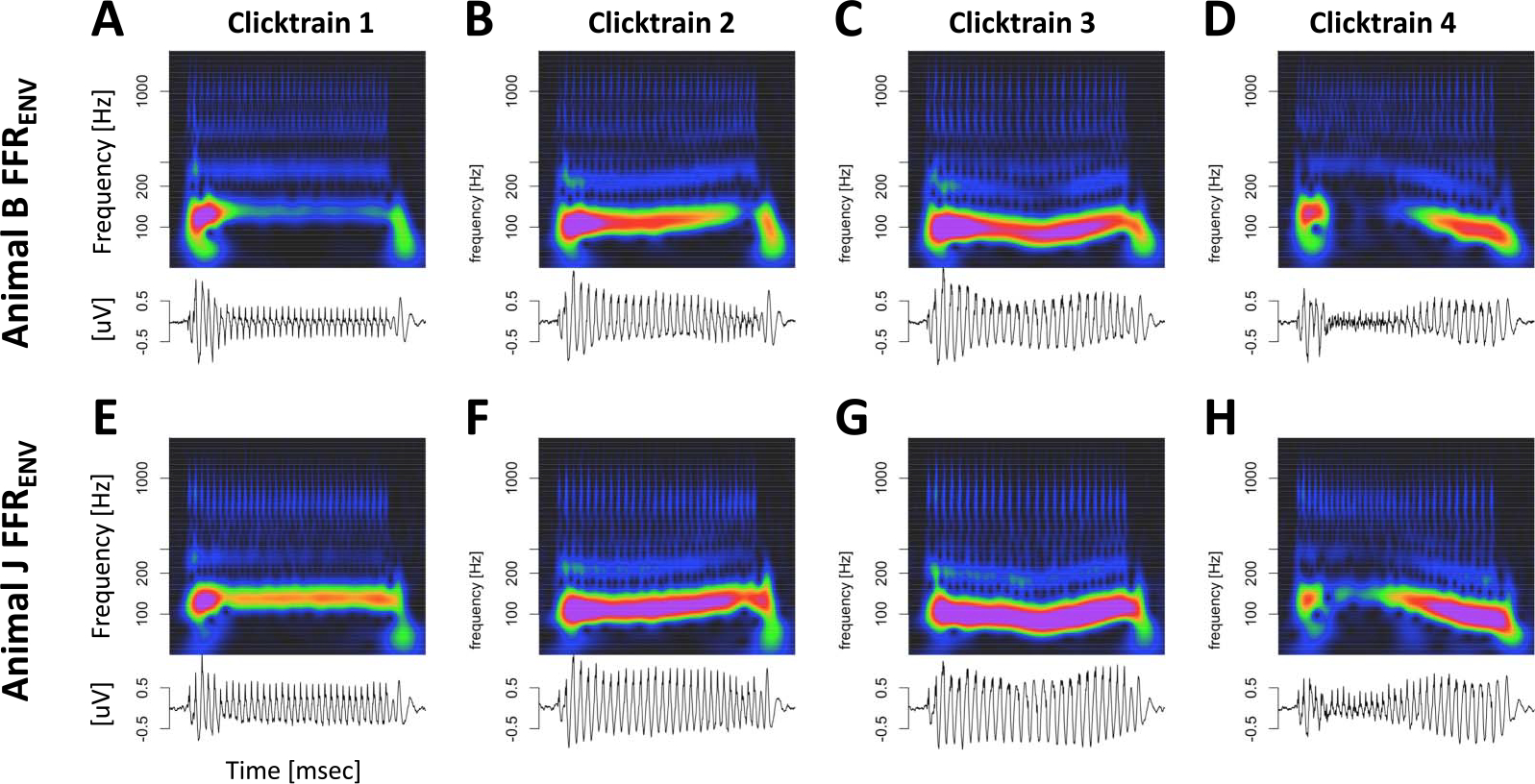
Click train FFR_ENV_. Representation of click train FFR_ENV_ in the time and time-frequency domains. (A–D) Monkey B click train FFR_ENV_. (E–H) Monkey J click train FFR_ENV_.

As expected, both types of stimuli elicited periodic FFR_ENV_-like responses in both animals. Figure 3 depicts the Mandarin tone stimuli as well as the grand average FFR_ENV_ in the time and time-frequency domains for both subjects. In the time domain, we observed a wide diversity of shapes of the FFR_ENV_ as F0 changed both within and between different Mandarin tone stimuli. In the time-frequency domain, we observed modulation of the fundamental frequency (F0) and the first harmonic (H1) in concert with the dynamically changing fundamental frequency of the Mandarin tone stimuli. Figure 4 depicts the click train FFR_ENV_ in the time and time-frequency domains. The click train FFRs were qualitatively similar, but of larger amplitude than the Mandarin tone FFR_ENV_. In the time-frequency domain, we observed power above the first harmonic. Especially for animal B, there was evidence of a second harmonic (F2) in cases when F0 was low, such as for click train #3 or toward the end of click train #4. Furthermore, we often observed power beyond the second harmonic in even higher frequency bands >400 Hz. In contrast to the first and second harmonic, the frequency of these higher-frequency components did not change in line with the fundamental frequency of the stimulus. These higher frequencies were also present for the tone FFR_ENV_, but harder to distinguish due to their lower amplitude. Based on the time-frequency decomposition of the FFR_ENV_, we will focus on three different frequency bands: the frequency range of the fundamental frequency F0 (70–170 Hz), the frequency range of the first harmonic H1 (180–300 Hz), and the frequency range beyond the second harmonic Hx (400–1200 Hz).

### Deconvolution of Click Train FFR_ENV_

We next set out to test if FFR_ENV_ with such a complex phenomenology both in the time and time-frequency domains can be explained by a simple linear superposition model. Given their larger amplitude and thus higher signal-to-noise ratio, we first focused on the click train FFR_ENV_. To further improve signal-to-noise ratio, we initially focused on data averaged across all recording sessions. To that aim, data from each session was randomly split into two equally sized sets, subsequently referred to as the training set and test set, respectively. Within each set, trials were averaged across the four different click train stimuli. The deconvolution was performed on the four click train FFR_ENV_ averaged across all training sets. The model fit was then evaluated by comparing the model predictions derived from the training set with the data from the testing set.

**Figure 5.**
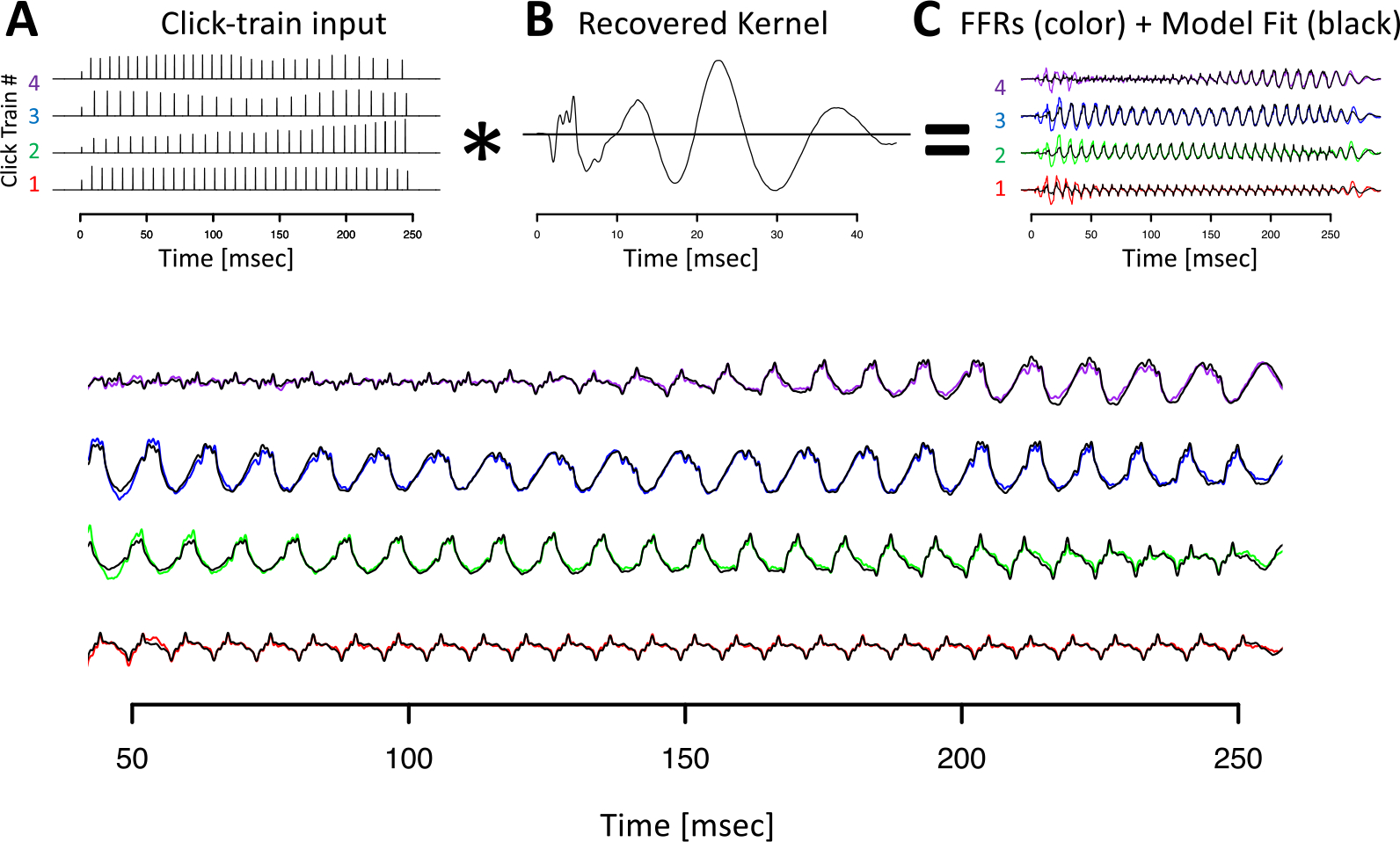
Deconvolution of grand average click train FFR_ENV_ for animal B. (A) Click train regressor for the four click train stimuli. The F0 contour of click train #1 matches the high tone, #2 the rising tone, #3 the dipping tone, and #4 the falling tone. (B) Recovered kernel which can be viewed as the impulse response to one click. (C) Observed click train FFR_ENV_ (color) and model fit (black). (D) Enlargement of the steady state period of the FFR_ENV_ response.

Figure 5 visualizes the deconvolution process, the F0_ENV_ response, also referred to as the FFR_ENV_ kernel, and the model fits in the time domain for animal B. All key features of the click train FFR_ENV_ were well-captured by the convolution model (black lines in Figure 5C, D). It is noteworthy that the wide range of shapes of the click train FFR_ENV_ could be accounted for with just one underlying kernel. The different shapes of the click train FFR_ENV_ were created exclusively by slight variations of constructive and destructive interference driven by subtle timing and amplitude differences from otherwise identical F0_ENV_ responses to individual clicks. In both animals, the extracted kernels contained two key spectro-temporal features: a series of brisk peaks and troughs with short latencies and high-frequency, as well as wavelet-like responses at longer latencies and a lower frequency (Figure 5B).

**Figure 6.**
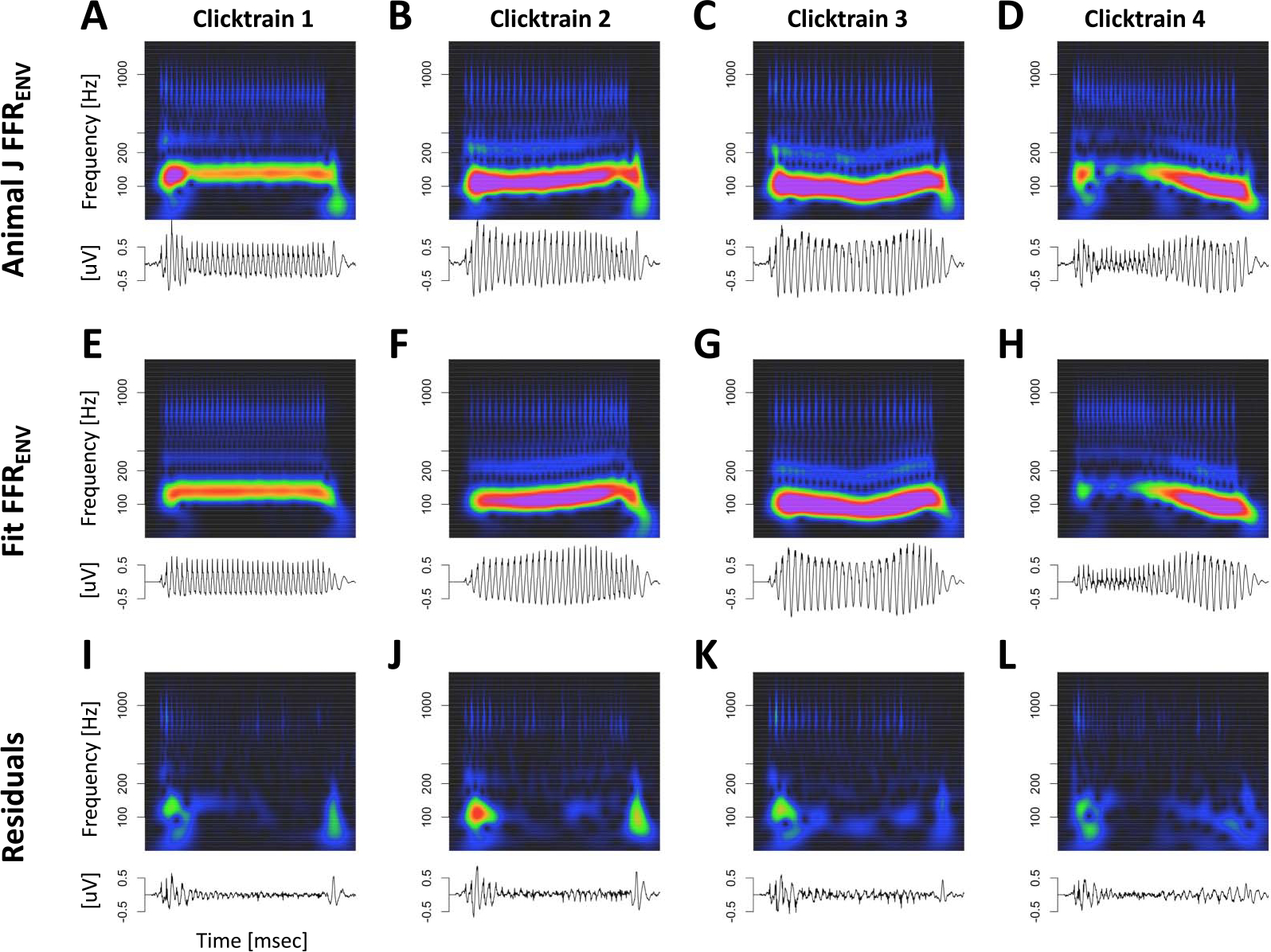
Deconvolution of grand average click train FFR_ENV_ for animal J in the time and time-frequency domains. (A–D) Click train FFR_ENV_. (E–H) Fit of the deconvolution model. (I–J) Residuals of the model fit.

Figure 6 visualizes the deconvolution process for animal J in the time and time-frequency domains. This visualization confirmed that the model captured key aspects in all relevant frequency bands and not just the fundamental frequency. Note that the model captured the components whose frequency changed dynamically with F0 (fundamental and first harmonic), as well as the higher frequency components above F2 whose frequency is unaffected by dynamic F0 of the stimulus (or the ensuing FFR_ENV_).

**Figure 7.**
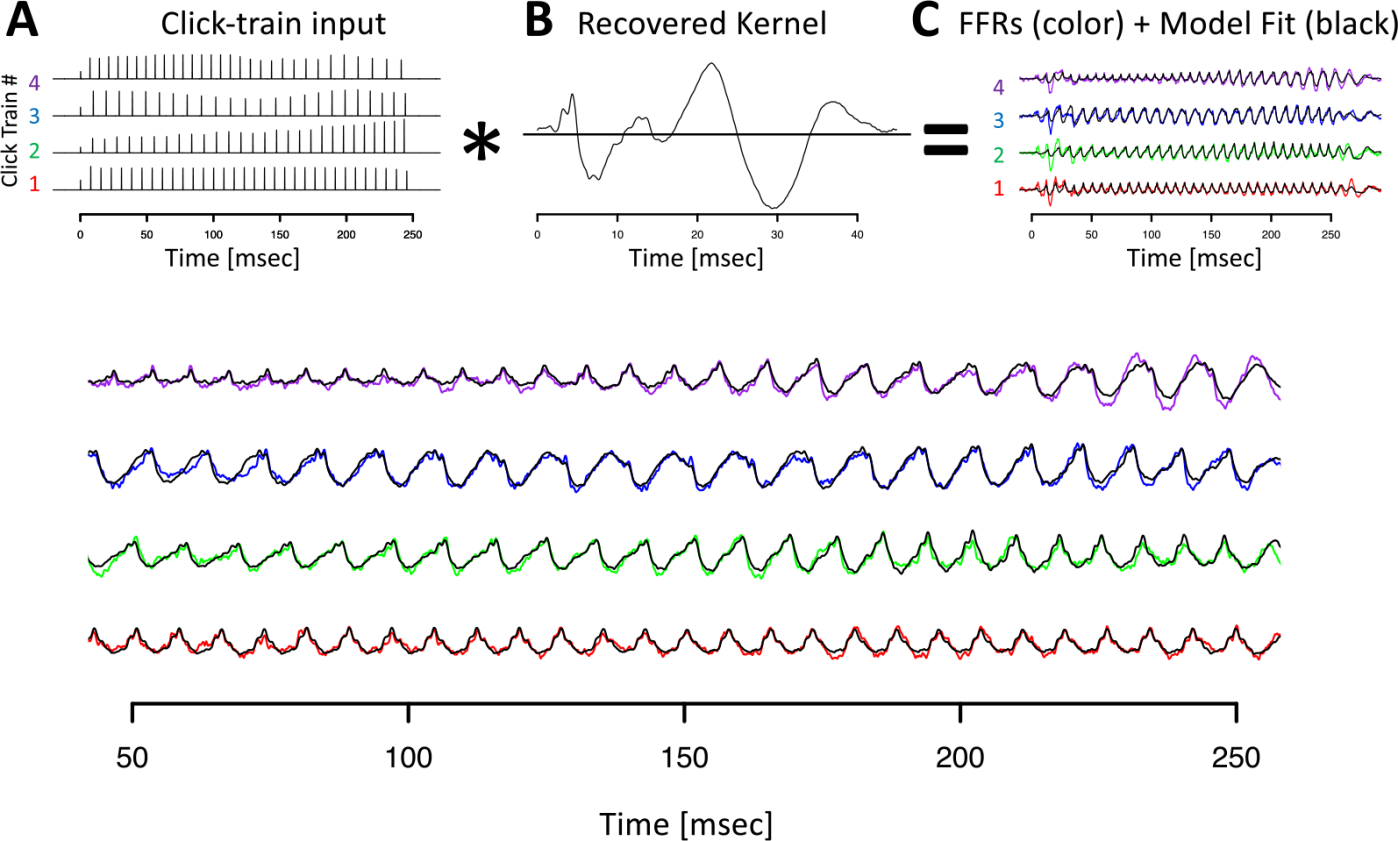
Deconvolution of grand average tone FFR_ENV_ for animal J. Conventions as in Figure 4. Note that the click trains in panel A refer to the predictors used in the deconvolution, not the Mandarin tone stimulus used to drive the FFR_ENV_.

Figure 7 visualizes the deconvolution process for the Mandarin tone stimuli in the time domain. Other than using FFR_ENV_ recorded in response to Mandarin tone, the procedure for obtaining the F0 kernels was identical, and the results closely resembled the ones obtained for the click train stimuli.

While the convolution model captured all key aspects of the data, we also observed regions of systematic deviations. In particular, the model underestimated the response amplitudes during the first ~50 ms of the stimulus. In part to compensate for this, the model tended to over-estimate the amplitudes for the remainder of the stimulus. This effect may likely be caused by short-term adaptation, a nonlinear effect that cannot be accounted for by a strictly linear model. We will briefly touch on this issue later in the article by introducing a nonlinear–linear convolution approach that resolves most of the remaining systematic misfit during the onset period.

### Percentage Variance Explained: Click Train FFR_ENV_

We next quantified the performance of the model as the percentage variance explained, either calculated across the entire stimulation period (0 to 280 ms after stimulus onset), or the sustained period which excluded both on- and offset responses (50 to 250 ms). Furthermore, we evaluated percentage variance explained in three different frequency bands, the frequency range of the fundamental frequency F0 (70–170 Hz), the frequency range of the first formant F1 (180–300 Hz), and the frequency range beyond the first formant Fx (400–1200 Hz). See Methods for details.

Because no model can be expected to account for measurement noise, percentage variance explained cannot exceed a threshold of 100 − 100/signal-to-noise ratio. As a result, the traditional metric of percentage variance explained is only comparable for data sets with similar signal-to-noise ratio. Thus, we decided to quantify how much of the “explainable” variance, i.e., the portion of the variance that exceeds the variance of the baseline, can be explained by the model. See Methods for details.

**Figure 8.**
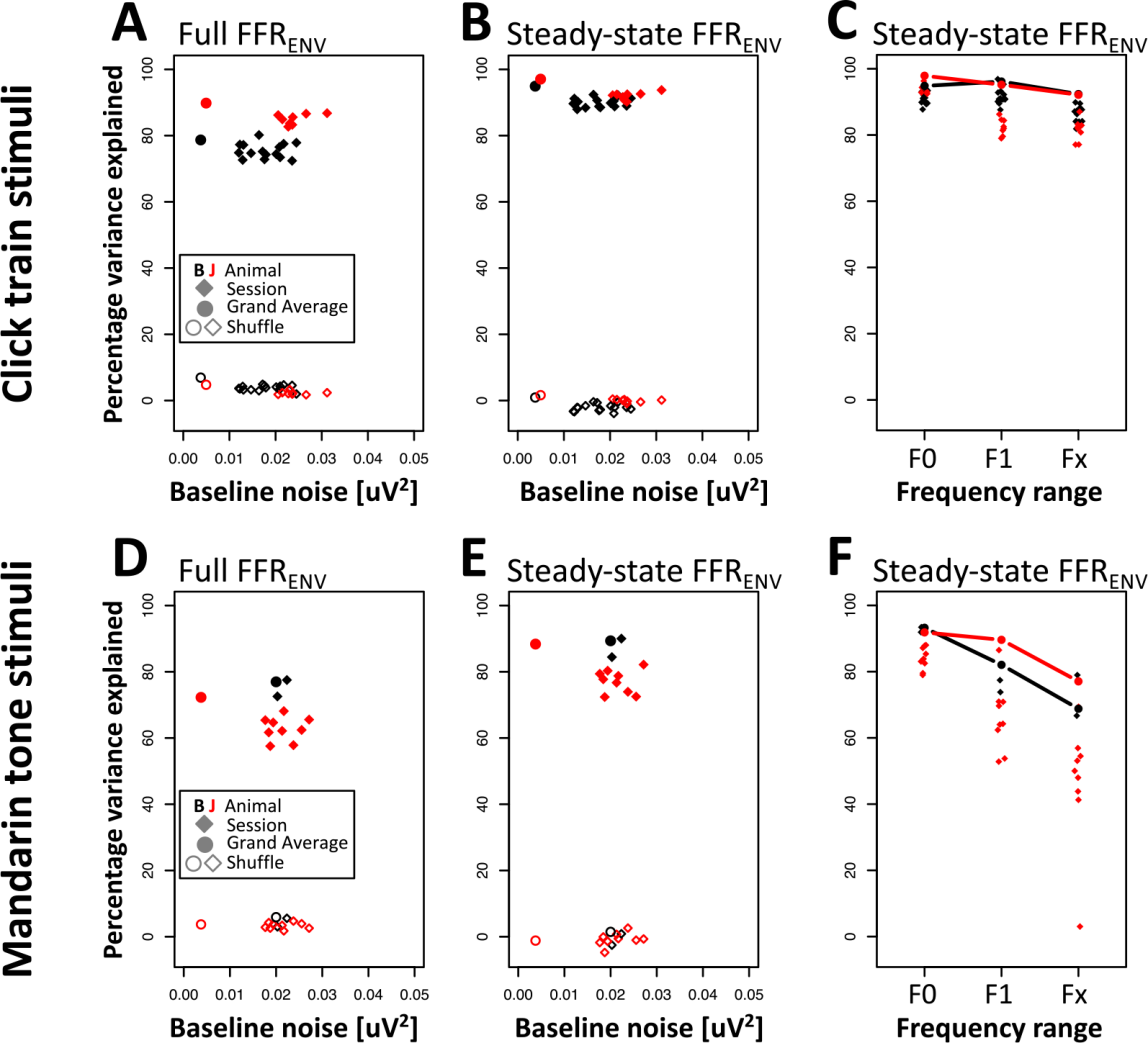
Percentage variance explained. (A) Percentage variance explained across the entire FFR_ENV_ as a function of baseline noise. Solid points indicate fits to the grand averages across all sessions. Solid diamonds indicate fits to individual sessions. Unfilled symbols indicate fits using shuffled predictors. (B) Same as (A) but percentage variance explained is only evaluated for steady state portion of the FFR_ENV_ (50–250 ms). (C) Percentage variance explained by frequency band. (D–F) same as (A–C) but for Mandarin tone stimuli.

In both animals, the convolution model explained the vast majority of the explainable variance (monkey B: 79%; monkey J: 90%, solid circles in Figure 8A). This value was even higher in the sustained period that excluded on- and offset responses (monkey B: 95%; monkey J: 97%; solid circles in Figure 8B). Within the sustained period, there was a gradient of percentage variance explained by frequency range. The largest fraction of variance could be explained in the F0 range, followed by the H1 and Hx ranges (F0 range: 95% and 98%, for monkey B and J, respectively; F1 range: 96% and 95%; Fx range: 92% and 92%, solid circles and lines in Figure 8C).

We next tested if the high percentage of explained variance was caused by overfitting. To that aim, we used a shuffle control in which the number of predictors remained constant but no longer matched the timing and amplitude of the actual F0 onsets (see Methods for details). This shuffling dramatically attenuated the percentage variance explained (animal B: 7%, animal J: 5%, open circles in Figure 8A). The percentage variance explained was even smaller in the sustained period (animal B: 1%, animal J: 2%, open circles in Figure 8B). The lower values for the sustained period likely occurred because the shuffled model tended to capture variance at stimulus onset (which is identical for all stimuli) at the expense of the sustained period.

We next set out to quantify how much of the click train FFR_ENV_ can be explained by the linear kernel in more common experimental settings, i.e., from data collected in individual recording sessions. To that aim, we calculated the kernel from data averaged across one recording session and evaluated the fit by comparing the predictions to the FFR_ENV_ of all other recording sessions. The results largely replicated the findings at the level of the grand averages and confirmed that a substantial amount of the explainable variance could be captured by the linear model even at the level of individual recording sessions (animal B: 75 ± 2.7%, animal J: 85 ± 2.7%, mean standard deviation, solid diamonds in Figure 8A). An even higher percentage of the variance was captured during the sustained period (animal B: 90 ± 4.0%, animal J: 92 ± 2.9%, solid diamonds in Figure 8B). Results from the shuffle control predictor confirmed that overfitting was also not a major concern for the single session data (animal B: 4 ± 0.9%, animal J: 2 ± 0.9%, open diamonds in Figure 8A). The percentage variance explained by the shuffle predictor was even smaller in the sustained period (animal B: −2 ± 1.3%, animal J: 0 ± 0.9%, open diamonds in Figure 8B). The negative values for animal B indicate that the shuffle predictor inflated the variance in the sustained period.

Furthermore, the single-session analysis confirmed that the model captured the most variance in the frequency range of the F0 (animal B: 91 ± 4.4%, animal J: 94 ± 3.3%, solid diamonds in Figure 8C), followed by the frequency range of the F1 (animal B: 92 ± 2.7%, animal J: 82 ± 4.8%), and the highest frequency range Fx (animal B: 86 ± 2.9%, animal J: 82 ± 4.8%).

### Percentage Variance Explained: Mandarin Tone FFR_ENV_

The results so far suggest that the deconvolution method works rather well on artificial click train stimuli. By itself, this is an important finding. However, given the substantial differences between click trains and speech, we then tested if the method also explains much of the variance of the FFR_ENV_ in response to the spectro-temporally complex and realistic Mandarin tones.

As for the click train stimuli, we first computed the deconvolution on data combined across all recording sessions for each animal. Kernels were fit to a training set and the quality of the fits were then evaluated by comparing the predictions to the FFR_ENV_ of the test set. In both animals, the convolution model explained a large proportion of the explainable variance (monkey B: 77%; monkey J: 72%, solid circles in Figure 8D). This value was even higher in the sustained period that excluded on- and offset responses (monkey B: 89%; monkey J: 88%, solid circles in Figure 8E). Within the sustained period, there was a clear gradient of percentage variance explained by frequency range. The largest fraction of variance could be explained in the F0 range, followed by the H1 and Hx ranges (F0 range: 93% and 92%, for monkey B and J, respectively; F1 range: 82% and 90%; Fx range: 69% and 77%, solid circles and lines in Figure 8F).

As for the click train stimuli, using the shuffled predictor dramatically attenuated the percentage variance explained (animal B: 6%, animal J: 4%, open circles in Figure 8D). The percentage variance explained was even smaller in the sustained period (animal B: 1%, animal J: −1%, open circles in Figure 8E).

Despite the overall lower signal amplitudes for the tone FFR_ENV_, a large proportion of the variance was captured by the linear convolution model even on a session-by-session basis (animal B: 75 ± 3.5%, animal J: 63 ± 4.0%, mean ± standard deviation, solid diamonds in Figure 8D). Excluding on- and offset responses, the percentage variance explained is even higher (animal B: 87 ± 3.9%, animal J: 77 ± 4.6%, filled diamonds in Figure 8E). As for the grand averages, shuffling dramatically attenuated the percentage variance explained at the single session level (animal B: 4.0 ± 1.8%, animal J: 3.0 ± 1.9%, open diamonds in Figure 8D; sustained period: animal B: −1 ± 2.4%, animal J: −1 ± 2.9%, open diamonds in Figure 8E), again confirming that overfitting was not a substantial contribution to the high percentage of variance explained.

Furthermore, the single-session analysis confirmed that the model captured the most variance in the frequency range of the F0 (animal B: 93 ± 1.1%, animal J: 84 ± 3.8%, solid diamonds in Figure 8F), followed by the frequency range of the F1 (animal B: 76 ± 2.5%, animal J: 66 ± 11.1%), and the highest frequency range Fx (animal B: 73 ± 8.7%, animal J: 47 ± 19.5%).

### Consistency of Deconvolution Approach Across Recording Sessions

**Figure 9.**
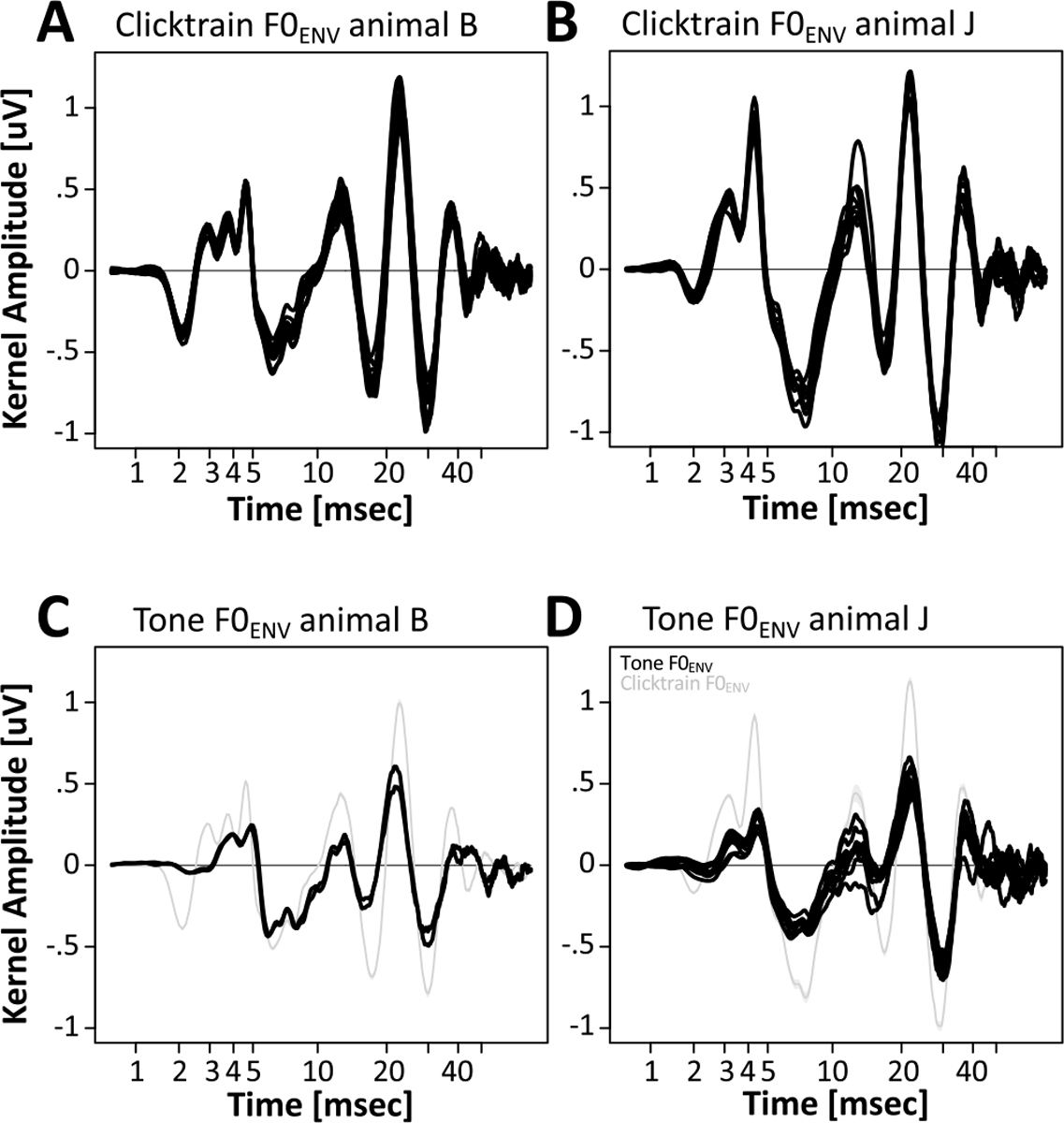
Comparison of F0_ENV_ responses across sessions, stimuli, and subjects. (A, B) Click train F0_ENV_ responses for individual sessions of animals B and J. (C, D) Mandarin tone F0_ENV_ responses for individual sessions of animals B and J.

The ability to explain the FFR_ENV_of one recording day using the kernel from a different session, suggests that the kernels are remarkably similar between days. Figure 9A, B confirms the high degree of similarity for the click train kernels. Especially early features of the kernel (<5 ms) were highly preserved across sessions, to the point that it was hard to even distinguish the presence of more than one trace. Above 5 ms, differences between sessions became somewhat more apparent. The largest between-session variability was observed for the late wavelet-like response between 15 and 35 ms. We quantified the similarity of the kernels as the Pearson correlation coefficient, which was found to be 0.97 ± 0.02 for both animals (mean plus minus standard deviation). Note that while the kernels for different sessions were highly similar, the kernels for the two animals were quite distinct from each other. In particular, the early features of the kernels below 5 ms are like a fingerprint that uniquely identifies the subject with high confidence on the basis of a single session.

Cross correlations for kernels of the Mandarin tone stimuli (Figure 9C, D) were similarly high (animal B: 0.98 ± NA, animal J: 0.91 ± 0.08; standard deviation was not available for animal B, since only two sessions were recorded, resulting in a single cross-correlation value. For monkey J, the average cross-correlation was attenuated mostly by one session. As a result of the leftward skew of the distribution, the median correlation coefficient was a good bit higher and probably a more robust estimate (median correlation coefficient monkey J: 0.95).

### Spectro-Temporal Features of the F0_ENV_ Responses

**Figure 10.**
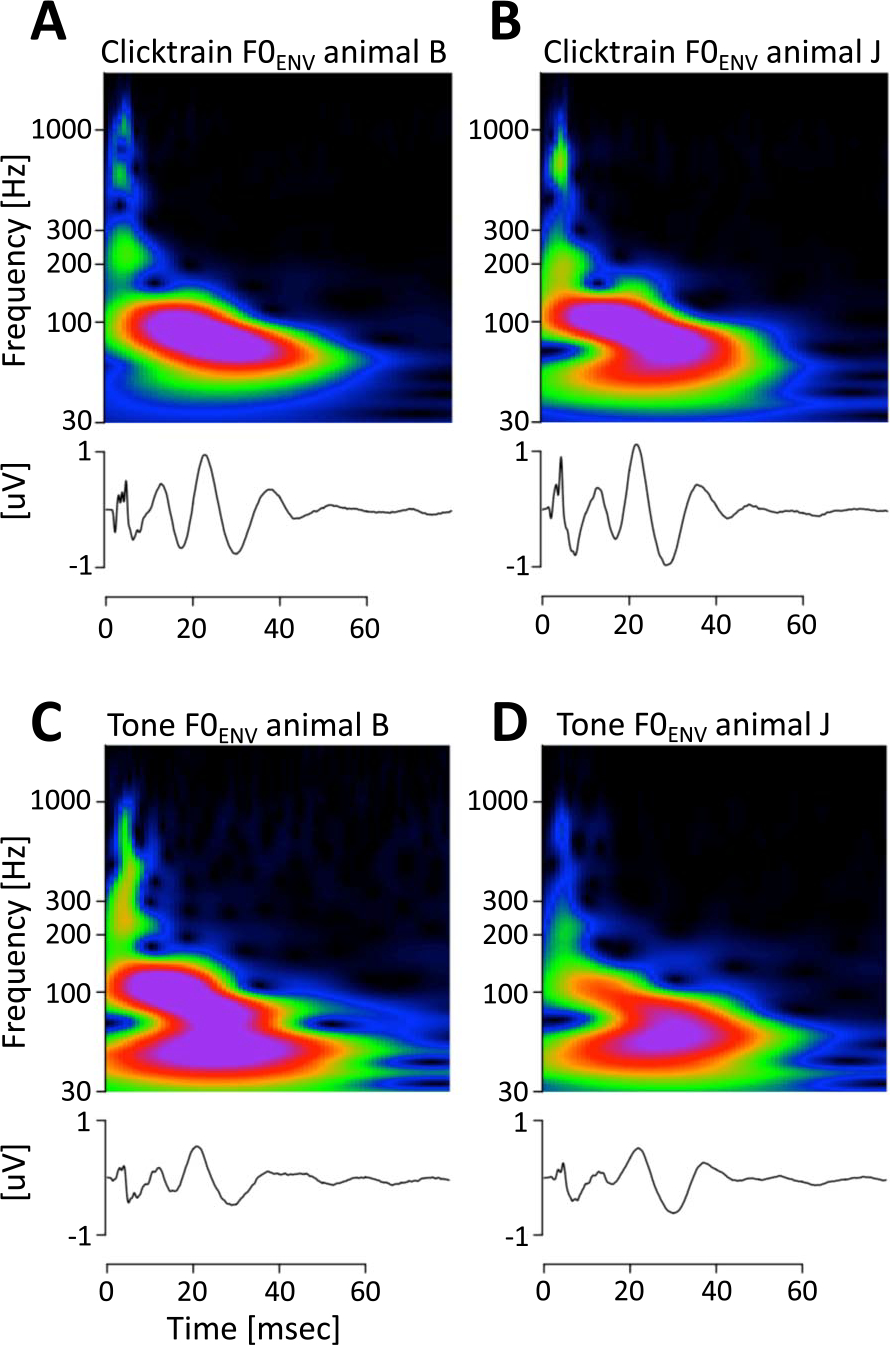
Comparison of F0_ENV_ responses across stimuli and subjects in the time-frequency domain. (A, B) Average click train F0_ENV_ responses for animals B and J. (C, D) Average Mandarin tone F0_ENV_ responses for animals B and J.

Based on the time domain description of the kernels, they could be split into three epochs: (1) a short-latency period from 1 to 5 ms that featured a series of brisk peaks and troughs; (2) a transition period at middle latencies from 5 to 15 ms; (3) a long-latency period from 15 to ~45 ms that featured 3 peaks and 2 troughs of a large amplitude and relatively slow, wavelet-like oscillation. In the short-latency period, both animals exhibited a prominent trough at ~2 ms and a prominent peak at ~4.5 ms. In between the two, animal B featured two peaks at 2.9 and 3.7 ms, while animal J featured only one intermittent peak at 3.1 ms. The peak at ~4.5 ms likely corresponds to wave V of the brainstem auditory evoked potential. Transforming the kernels into the time-frequency domain revealed a complex spectral composition that confirmed the notion of distinct periods in the kernel (Figure 10A, B, top panels). At short latencies, both animals exhibited prominent high-frequency components above 500 Hz: In animal B, they manifested in two distinct spectral peaks at 600 and 1050 Hz. In animal J, they manifested as a single peak at 700 Hz. In addition, both animals show spectral power at frequencies around 200 Hz. For both animals, activity in this frequency range extended into the middle latency period. The key spectro-temporal feature of the kernel was an extended period of power in the lower frequency range between 70 and 120 Hz. Closer inspection revealed a gradual decrease of frequency over time: In animal B the frequency decreased from 90 Hz to 70 Hz, in animal J the frequency decreased from 105 to 75 Hz. It is unclear if this decrease resulted from the gradual change of frequency of a single component, or from the transition between two components with slightly different frequencies.

The detailed description of the kernels in Figures 9 and 10 enables a direct comparison of the tone and click train kernels. The most striking difference is the overall reduced amplitude of the kernels, which matches the overall reduced amplitudes of the tone FFR_ENV_ themselves (Figure 9). However, most of the key features of the kernels were preserved. In particular, the timing and polarity of most peaks were identical. Only the earliest putative brainstem components were affected more strongly. In both animals the initial trough that was evident at ~2 ms for the click train kernels was reduced in amplitude, temporally smeared and delayed to ~3 ms. In animal B, this temporal smearing may have contributed to the cancellation of the first of the three subsequent positive peaks that occurs at 2.9 ms in the click train kernel. Figure 10 highlights another interesting distinction that is not visible in the time domain. For both animals, the tone kernels included power in an even lower frequency band centered around 50 Hz that was not active for the click train kernels.

### Topography of the Click Train F0_ENV_ Responses

**Figure 11.**
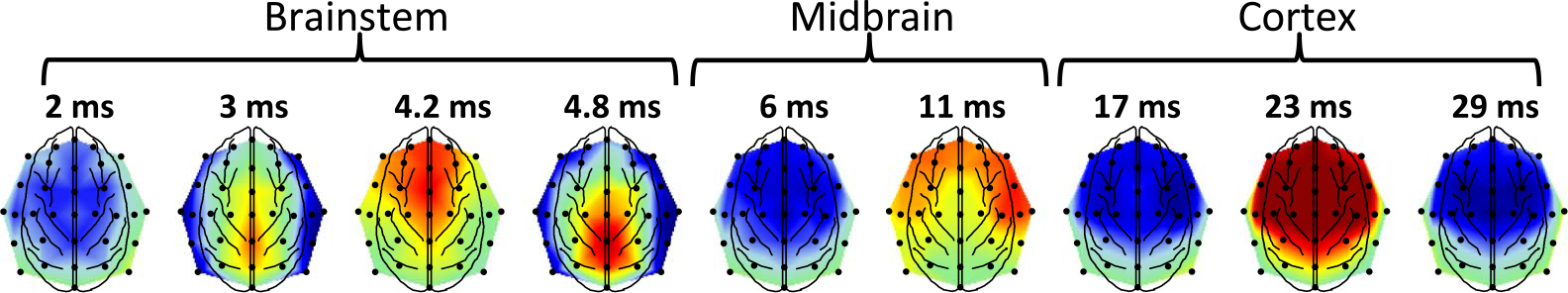
Topography of click train F0 responses. Topography of different peaks and troughs of the F0 onset response for animal B. Different components are tentatively grouped into brainstem, midbrain, and cortex based on latency, frequency, and topography.

It is tempting to link these different spectro-temporal features of the kernel to processing in brainstem, midbrain, and cortex, respectively. If correct, it would support the notion that the deconvolution method was indeed able to partially disentangle these different generators whose activity is temporally completely overlapping in the FFR_ENV_. If different latencies of response components in the FFR_ENV_ kernel indeed reflect the gradual activation of successively higher stages of auditory processing, then this should be reflected in different topographies for early relative to late components. In one subject, animal B, we had access to an entire grid of 33 EEG electrodes. We thus set out to estimate the kernels for all 33 EEG electrodes in this animal. The resulting topographies are summarized in Figure 11. The topographies of the putative cortical components indeed closely resembled the topographies of classical evoked potentials that are believed to arise from core auditory regions in the superior temporal plane (Teichert, 2016). In contrast, the putative brainstem topographies were much more varied, and, except for the peak at 4.2 ms, clearly not of cortical origin. The topographies of the putative midbrain components were diverse. While the topography of the component at 6 ms was not unlike the classical cortical topography, the component at 11 ms was clearly not suggestive of cortical origin.

### Nonlinear–Linear Deconvolution Model

For both stimulus types, the linear model could predict a surprisingly large amount of the variance. However, in both cases, even the click train FFR_ENV_, the linear model fell short of explaining a substantial amount of variance around stimulus onset. The observed pattern of misfit suggests that short-term adaptation prevents the linear model from providing an even better account of the data. To confirm this hypothesis, we developed a two-stage model that includes a nonlinear first stage to account for short-term adaptation, and the linear convolution model as a second stage. The short-term adaptation model uses two parameters, tau and U, to estimate how quickly and how strongly early stages of the auditory system adapt to the repeated F0 onsets. In addition, the model included a stimulus onset regressor. To keep the total number of regressors comparable, we reduced the number of lags from 800 to 450 for both regressors (see Methods for details).

In both animals, the nonlinear–linear convolution model improved model fits for the click train stimuli, especially in the onset period (monkey B: 58% to 91%, monkey J: 72% to 92%). Noticeable improvements could also be found when focusing on the entire FFR_ENV_ (monkey B: 79% to 92%; monkey J: 90 to 94%). Importantly, percentage variance improved or remained constant even in the sustained period (monkey B: 95% to 97%; monkey J: unchanged at 97%), even though fewer degrees of freedom were used to model the sustained period (rather than 800 parameters, the nonlinear–linear model used only two nonlinear parameters plus 450 F0_ENV_ response parameters to model the sustained period; the 450 predictors for stimulus onset have no direct effect on the sustained period). Similar improvements were found for the Mandarin tone stimuli in the onset period (monkey B: 55% to 90%, monkey J: 42% to 91%), across the entire FFR_ENV_ (monkey B: 77% to 88%, monkey J: 72% to 87%), and in the sustained period (monkey B: 89% to 92%, monkey J: 88% to 88%).

The time constants tau of the short-term synaptic depression that provided the best fit were well below 100 ms for the click train stimuli (monkey B: 63 ms, monkey J: 26 ms) and the Mandarin tone stimuli (monkey B: 74 ms, monkey J: 13 ms). Such short time constants are consistent with a locus of adaptation in the early auditory system.

### Temporal Fine Structure of Mandarin Tone FFR

The analyses so far have focused exclusively on the FFR_ENV_. In the following we will focus on the temporal fine structure of the FFR, or FFR_TFS_, which is highlighted by subtracting the averages of the two polarities.

**Figure 12.**
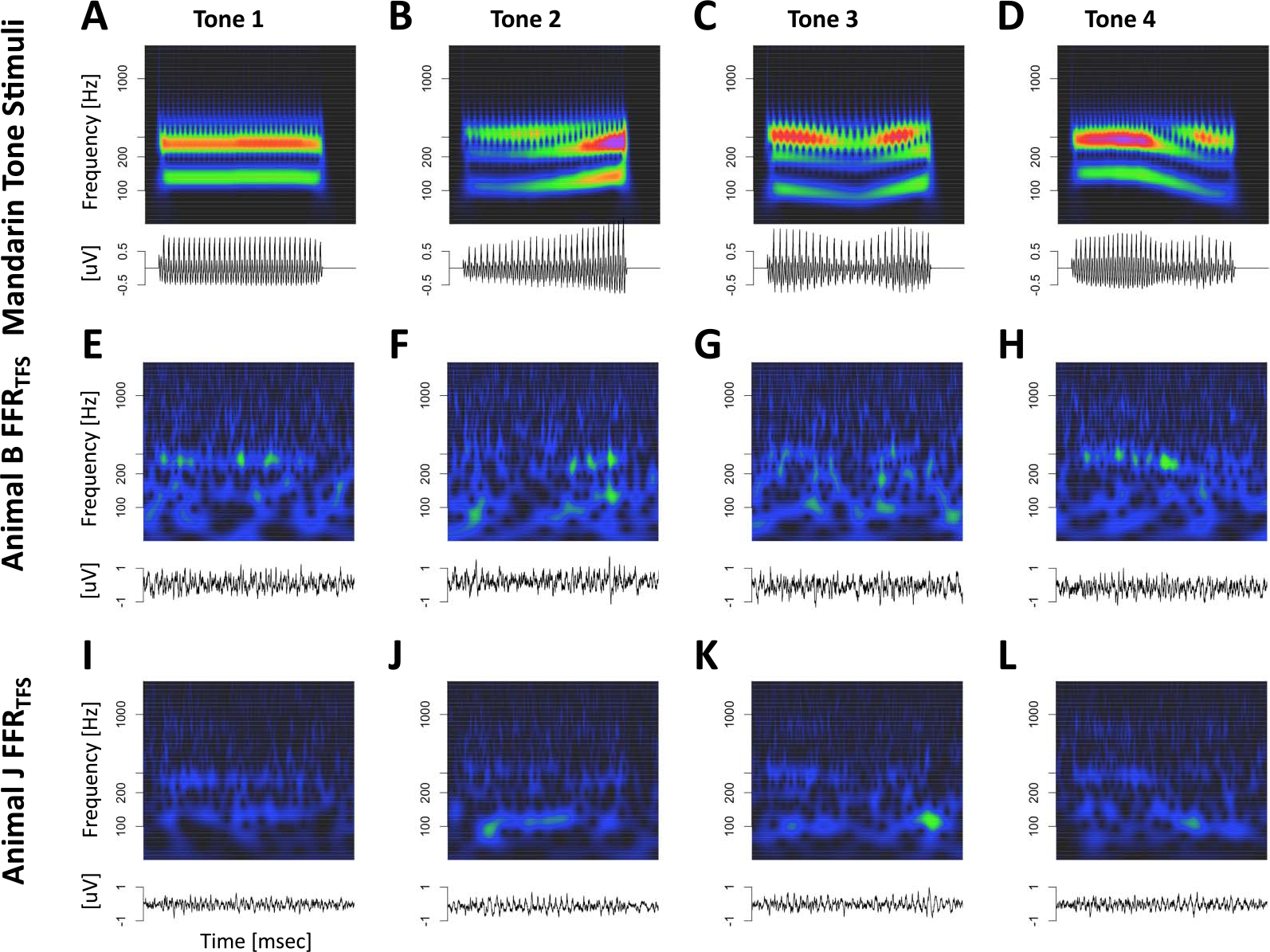
Mandarin tone FFR_TFS_. (A–D) Representation of Mandarin tone FFR_TFS_ in the time and time-frequency domains. (E–H) Monkey B click train FFR_TFS_. (I–L) Monkey J Mandarin tone FFR_TFS_.

Figure 12 displays Mandarin tone FFR_TFS_ for both animals in the time and time-frequency domains. FFR_TFS_ for the click train stimuli were so small that we did not attempt to model them with the deconvolution approach. Compared to FFR_ENV_, FFR_TFS_ showed weaker responses to the fundamental frequency, thus highlighting responses to higher harmonics.

**Figure 13.**
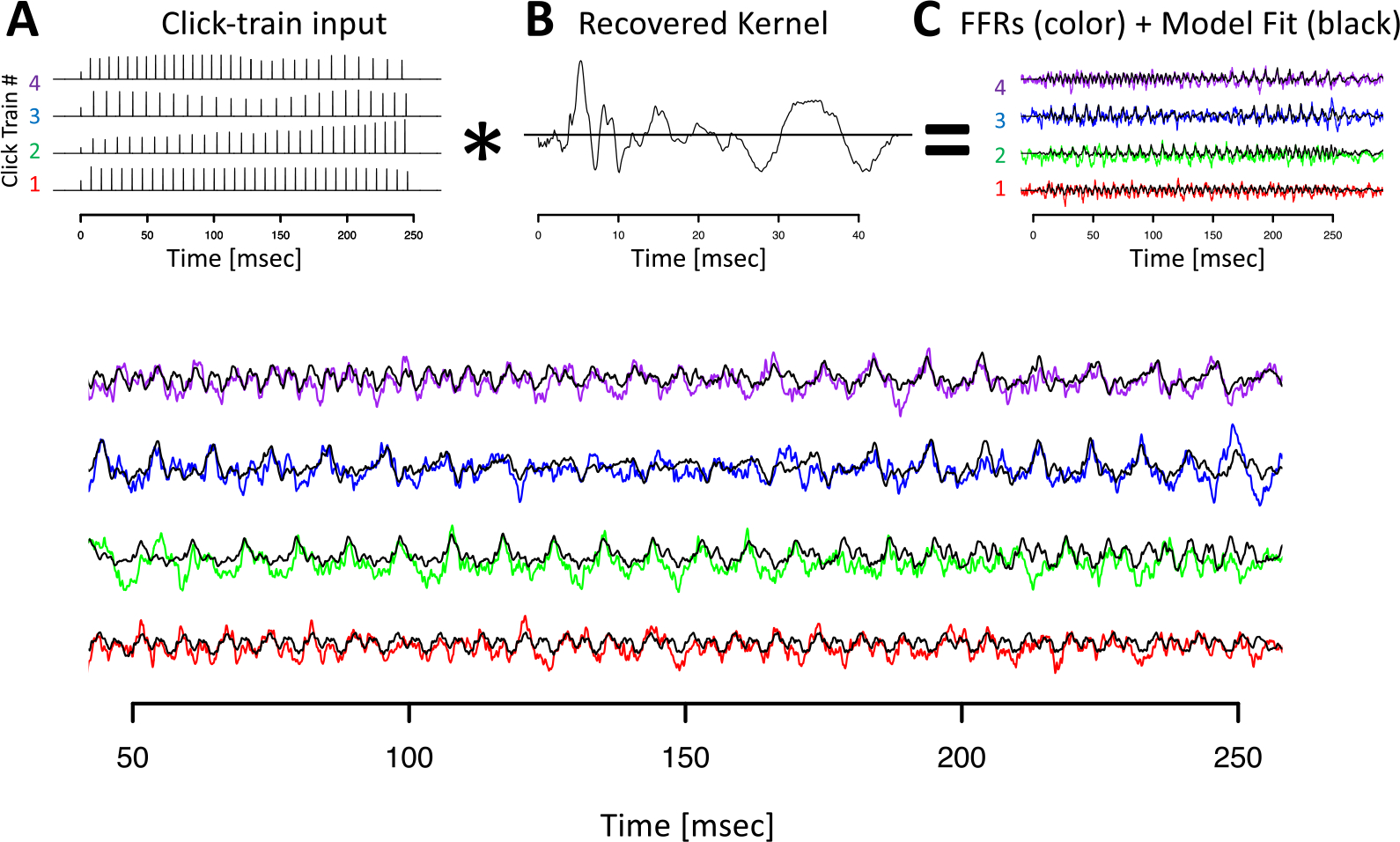
Deconvolution of grand average tone FFR_TFS_ for animal J. Conventions as in Figure 7.

**Figure 14.**
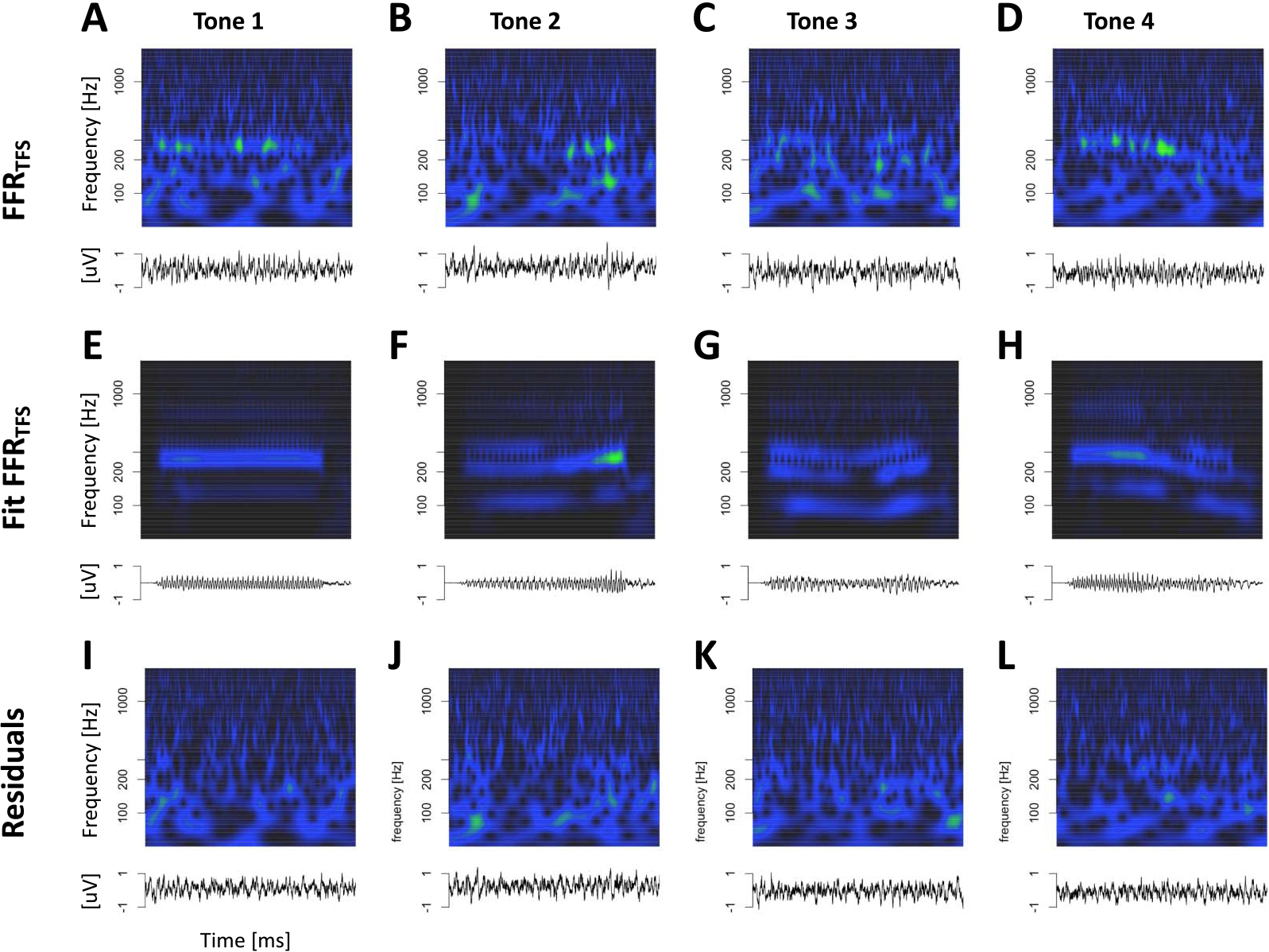
Deconvolution of grand average tone FFR_TFS_ for animal B in the time and time-frequency domains. (A–D) tone FFR_TFS_. (E–H) Fit of the deconvolution model. (I–J) Residuals of the model fit.

Despite the different theoretical interpretation of the FFR_TFS_, it can readily be modeled using the same deconvolution approach. Figure 13 shows the deconvolution process for FFR_TFS_ to Mandarin tone stimuli in one example animal. Figure 14 visualizes the fitting process in the time and time-frequency domains for the second animal. Because of the lower signal-to-noise ratio of FFR_TFS_, the correspondence between data and model is not as clear as for the FFR_ENV_. Nevertheless, the model correctly captures the fact that FFR_TFS_ contains power mostly in the range of the first formant, rather than the F0 as is the case for the FFR_ENV_. A second key observation is that most of the power of the FFR_TFS_ kernel is centered at relatively short latencies between 5 and 10 ms. This is a clear deviation from the FFR_ENV_ kernels that contained most of their power at latencies between 15 and 35 ms.

### Percentage Variance Explained: Mandarin Tone FFR_TFS_

Because FFR_TFS_ have a substantially lower signal-to-noise ratio, it is not surprising that the deconvolution model also explained a substantially lower percentage of the total variance. However, even when correcting for the lower signal-to-noise ratio, the model explained a substantially lower fraction of the explainable variance (monkey B: 38%; monkey J: 35%). Interestingly, the deconvolution approach captured a clearly distinct pattern of variance. First, in contrast to the FFR_ENV_, the model provided a better fit to the Mandarin tone rather than the click train FFR_TFS_ (click train data not shown). Second, in contrast to the FFR_ENV_, we observed only a negligible improvement when restricting our analysis to the sustained portion of the response (monkey B: 44%; monkey J: 36%). This is consistent with the notion that the onset responses, which complicate the analysis of the FFR_ENV_, are subtracted out for FFR_TFS_. Finally, we observed the highest percentage variance explained for the F1 rather than F0 frequency range as was the case for FFR_ENV_ (F0 range: 44% and 57%, for monkey B and J, respectively; F1 range: 85% and 73%; Fx range: 12% and 18%). Note that the F1 values are surprisingly high. This suggests that the decent quality of the fits is somewhat obscured by noise in the frequency ranges above and below the F1. The massive drop in performance for Fx range is consistent with the notion that auditory nerve cells cannot follow carrier frequencies above a certain limit.

### FFR_TFS_ Kernels (F0_TFS_ Responses)

**Figure 15.**
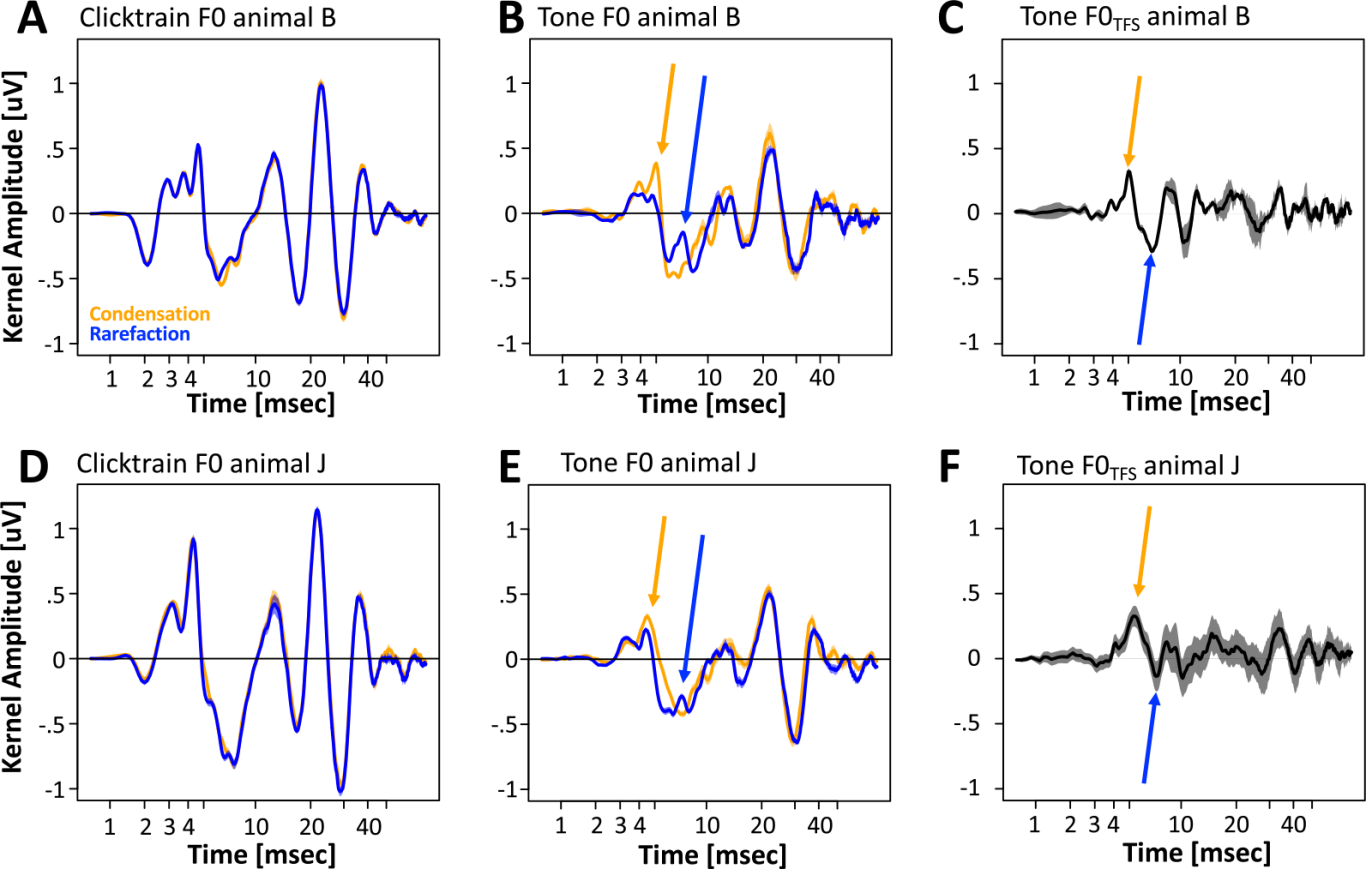
Effect of stimulus polarity on F0 responses. (A, B) Effect of stimulus polarity on click train kernels for monkeys B and J (orange: condensation, blue: rarefaction). (C, D) Same for Mandarin tone kernels.

Because the deconvolution operation is linear, the FFR_TFS_ kernels correspond to the difference of the kernels for the two different polarities (Figure 15). For the click train stimuli, the two polarities were quantitively almost identical, except for a minor deviation at a latency of ~7 ms. Note that while the effect was extremely small in absolute terms, it was replicable between sessions and present in both animals.

A qualitatively similar, but substantially larger effect emerged for the tone stimuli: The difference between the two polarities was most evident in the late brainstem and early midbrain latencies. In both animals, the putative component V of the brainstem response was strongly attenuated in the rarefaction condition (Figure 15B, E, orange arrow). In its stead, a new peak at a latency of ~7 ms that was superposed over the trough was also observed at this latency (Figure 15B, E, blue arrow).

## DISCUSSION

In this study, we characterized a deconvolution approach to recover F0_ENV_ and F0_TFS_ responses from FFR_ENV_ and FFR_TFS_ elicited by stimuli with time-varying pitch in the non-human primate. Our ultimate goal is to link pathologically altered FFR_ENV_ or FFR_TFS_ to specific latencies of the corresponding F0_ENV_ and F0_TFS_ responses and thus to narrow down their anatomical substrate. Such an approach would be particularly useful in clinical settings that often derive FFR_ENV_ and FFR_TFS_ with a simple three-electrode montage (Bidelman, 2015), and are thus not amenable to sophisticated source reconstruction analyses.

The most promising advances were made for the FFR_ENV_. First, we were able to show that the convolution model captures a substantial portion of the variance of the Mandarin tone and click train FFR_ENV_. Second, we were able to show that the kernels indeed have distinct spectro-temporal features that emerge at distinct latencies and likely reflect the sequential activation of generators in brainstem, midbrain, and cortex. Third, we were able to show that the FFR_ENV_ kernels can be estimated with high signal-to-noise ratio. Lastly, we were able to show that the method also works for FFR_TFS_, and that the resulting kernels have most power at middle latencies, consistent with sources in the midbrain. In the following we will discuss the implications of these advances in more detail.

### F0_ENV_ Onset Response Captures Much of the Variance of Mandarin Tone and Click Train FFR_ENV_

A key novelty of our study is that it allowed us to quantify how much variance of the FFR_ENV_ can be explained by the F0_ENV_ responses. This is important, because it determines the likelihood that the approach will be able to account for altered FFR_ENV_ in future work. To clarify why this is so important, we point out that the convolution approach can be viewed as data compression algorithm: Complex and high-dimensional FFR_ENV_ consisting of ~12,000 data points (4 tones times ~300 ms duration times 10 samples per ms) are represented by a much simpler kernel consisting of 800 data points (80 ms duration times 10 samples per ms). As with any data-compression algorithm, and especially for one with such a high compression ratio, its utility is determined by the amount of information loss. The less variance the algorithm captures, the more likely is a scenario where FFR_ENV_ differ meaningfully between conditions but the F0_ENV_ responses do not, simply because the relevant features of the FFR_ENV_ were not captured by the linear model.

In the best-case scenario, i.e., when excluding on- and offset responses and when using high signal-to-noise grand averages, the F0 responses can account for an average of 96% of the variance of the click train FFR_ENV_ and for 88% of the variance of the tone FFR_ENV_. Even at the level of single sessions, the model was able to explain on average 91% of the variance for the click train FFRs and 82% of the variance for the tone FFR_ENV_. Our finding that such a substantial portion of the FFR_ENV_ was explained by the convolution method increases the odds that F0_ENV_ responses will be able to capture many clinically relevant FFR_ENV_ phenomena. Since the F0_ENV_ responses capture more variance for the click train FFR_ENV_, one could argue in favor of using the click train stimuli in clinical settings. However, this would only be warranted if the click train FFR_ENV_ can be shown to be equally sensitive to pathological changes as other commonly used FFR_ENV_ stimuli.

It is worth noting that the F0_ENV_ responses are less adept at capturing variance in the higher frequency ranges. This drop-off is particularly pronounced for the Mandarin tone stimuli and for single sessions (rather than grand averages). Based on the observed latencies of features in the F0_ENV_ responses, the higher frequencies are likely generated at short latencies, i.e., by generators in brainstem. It is known that the latency of brainstem responses changes with sound intensity. Such changes of latency cannot be captured by the linear deconvolution model and may thus contribute to the reduction in percentage variance explained. Due to this and potentially other nonlinearities, the sensitivity of the linear deconvolution method will likely be reduced for pathologies in brainstem. However, it should be possible to capture such well-known nonlinearities by adjusting the nonlinear–linear model described above.

### F0_ENV_ Responses Compress FFR_ENV_ Into a Meaningful Format

We were also able to address a second key question that determines the utility of the deconvolution approach, namely whether or not the F0_ENV_ responses represent information about the FFR_ENV_ in a meaningful format. Specifically, we had speculated that the latency of different features of the F0_ENV_ response would represent the latency of different neural generators being activated sequentially along the ascending auditory hierarchy. Indeed, we were able to identify distinct spectro-temporal features that emerge at distinct latencies and likely reflect the sequential activation of generators in brainstem (<5 ms; 400–1000 Hz), midbrain (5–15 ms; 180–300 Hz), and cortex (15–45 ms; ~90 Hz).

This hypothesis was supported by two observations. First, the putative brainstem component of the F0_ENV_ responses very closely resembles actual brainstem responses recorded in response to individual clicks. In fact, the responses to the very first click of each click train (Figure 4C) showcase the exact same pattern and latencies of peaks and troughs as the F0_ENV_ responses of the click trains. Second, a cortical origin of the long-latency components is supported by distinct topographies (Figure 10) and direct intracranial recordings in primary auditory cortex of the monkey (Gnanateja et al., 2021). This leaves without strong empirical support only the putative midbrain components of the F0_ENV_ response. To date, we have not yet confirmed their putative origin using invasive recordings, but are planning to do so in the near future. These studies should be particularly relevant given the dominant role of the putative midbrain components of the F0_TFS_ response to the FFR_TFS_ (Figure 15). Given the well-established role of the midbrain in FFR_ENV_ and FFR_TFS_ in general (Chandrasekaran & Kraus, 2010; Greenberg et al., 1987; Smith et al., 1975), it would be surprising to find that the midbrain does not contribute to the F0_ENV_ response at all, or that it contributes at latencies other than the expected mid-latency range.

Our results are consistent with and extend some closely related earlier studies. Bidelman (2015) tested if the FFR_ENV_ to a click train stimulus can be explained as the superposition of empirically measured 12 ms long auditory brainstem responses to each click in the train. The conclusion from that article was that the FFR_ENV_ was not satisfactorily explained by auditory brainstem responses, suggesting that other structures must contribute to the FFR_ENV_. Our results are consistent with this conclusion. In order to explain the FFR_ENV_ well, it was necessary to allow the kernel to be at least 45 ms long, thus extending well beyond the temporal range of auditory brainstem latencies. Our results are also consistent with an earlier study showing that the auditory steady state response can be modeled as the linear superposition of onset responses to each individual 40 Hz cycle (Bohórquez & Özdamar, 2008). Our findings extend this work into a higher frequency range and into the realm of spectro-temporally complex speech sounds. More recent work, conducted in parallel with studies reported here, has used a similar deconvolution approach to calculate the F0 response from continuous speech (Polonenko & Maddox, 2021). In line with our findings, they also identified F0 responses that are consistent with the notion that they result from the sequential activation of generators along the ascending auditory pathway. Our work extends their findings by showing that F0_ENV_ responses account for the bulk of the FFR_ENV_ and likely also speech-evoked responses in general. In addition, our results point out the limitations of the linear superposition approach and how to address them by including a simple short-term adaptation component that adjusts the effective amplitudes of the F0 cycles.

### F0_ENV_ Responses Can Be Measured With High Signal-to-Noise

Finally, we were able to show that the F0_ENV_ responses can be estimated with high signal-to-noise ratio. The mean pairwise correlation coefficient between F0 responses estimated on different days was above 0.90 for both animals and both stimulus types. Such a high signal-to-noise ratio is possible because F0_ENV_ response is estimated from approximately 120,000 F0 cycles (4,000 trials, each of which contains on average 30 F0 cycles). The high signal-to-noise ratio of the F0_ENV_ responses suggest that even small effects can be detected with a very reasonable number of sessions or subjects and may thus provide a solid basis for downstream statistical inference.

### Comparison Between FFR_ENV_ and FFR_TFS_

While there are several reports of FFR_ENV_ in the monkey, our study is the first to report FFR_TFS_ in this species. Two key observations stand out. First, the signal-to-noise ratio of FFR_TFS_ is much smaller than FFR_ENV_. Second, the FFR_TFS_ was much weaker for the click train compared to the Mandarin tone stimuli. This finding is consistent with the notion that FFR_TFS_ reflects neural responses to individual cycles of relatively low frequencies, which are much more pronounced for the Mandarin tone compared to the click train stimuli.

FFR_TFS_ and FFR_ENV_ are typically believed to arise from rather distinct neural mechanisms. The simplifying assumptions of the deconvolution model are arguably less appropriate for the FFR_TFS_. Nevertheless, the deconvolution method captured important aspects of the FFR_TFS_, and confirmed several established differences between the FFR_TFS_ and FFR_ENV_. This increases our confidence both in the model system and in the utility of the deconvolution method. For example, the recovered FFR_TFS_ kernels had most of their power in a rather narrow middle latency range, thus suggesting an anatomically less diverse array of generators in the midbrain. This contrasts with the more wide-spread range of latencies of the FFR_ENV_ kernels.

Furthermore, the spectral power of the FFR_TFS_ was more closely linked to the spectral power of the stimulus. Specifically, both stimulus and FFR_TFS_ have most power in the intersection of the first and second harmonic with the first formant. In contrast, FFR_ENV_ has most power in the fundamental frequency, and higher harmonics are not modulated in line with the formants. The FFR_ENV_ contains substantial power in the Fx range, even though the stimulus itself has no power in that band. In summary, the spectral content of the FFR_ENV_ is mostly determined by the spectral content of the kernel, while the spectral content of the FFR_TFS_ is mostly determined by the stimulus.

### Limitations of the Linear Convolution Model

The high degree of variance that can be captured with the F0_ENV_ responses suggests that the neural responses to each click in the click train were able to propagate through subsequent stages of the auditory processing hierarchy largely without interference from previous or subsequent clicks that were being processed at the same time in higher or lower processing stages. Given the rich recurrent connections between different stages of the auditory hierarchy, and numerous well-established nonlinearities at the earliest stages of auditory processing (Dau, 2003; Heinz et al., 2001; Zilany et al., 2014), one might have predicted that a linear convolution model would be sorely insufficient to capture much of the spectro-temporal complexity of the FFR_ENV_.

However, it is also important to keep in mind that the linear model fell short of capturing all of the variance, especially around stimulus onset. Accounting for stimulus onset with an additional onset regressor and allowing the amplitudes of the click responses to be subject to short-term adaptation were able to increase percentage variance explained to above 90% even in the onset period. These results show that relatively minor deviations from the assumption of linearity can lead to substantial additional improvements.

Furthermore, it is important to mention that the deconvolution model explained substantially less variance for the FFR_TFS_. This likely reflects the lower signal-to-noise ratio of the FFR_TFS_ data, as well as the fact that the simplifying assumptions of the deconvolution model are less in line with the neural mechanisms generally believed to underly the FFR_TFS_.

### Future Directions

While the results so far are promising, several additional steps need to be taken before the method can be used to identify which processing stages are the cause of altered FFR_ENV_ and FFR_TFS_. Most importantly, the findings need to be confirmed in humans. Our own preliminary results as well as work with continuous “peaky” speech (Polonenko & Maddox, 2021) suggest very similar effects in humans. But the percentage of variance that is captured by the F0_ENV_ and F0_TFS_ responses remains to be determined for human participants. Furthermore, it is likely that at least initially latency by itself is not sufficient to unequivocally identify an underlying generator. Even for extremely well-established classical onset responses such as the different auditory brainstem response waves or the different mid-latency components, there is considerable debate about their more fine-grained origin. Consequently, we propose that the method should initially be calibrated in a sample data set with high-density EEG/MEG recordings to leverage both latency and topography of the F0_ENV_ and F0_TFS_ responses. Once the origin of different peaks and troughs has been established, subsequent analyses will be less reliant on high-density EEG recordings.

Furthermore, the ability of the deconvolution approach to correctly identify generators based on the latency of the F0_ENV_ and F0_TFS_ responses needs to be validated empirically by recording FFR_ENV_ and FFR_TFS_ directly from these structures. Published work from our group has already taken advantage of invasive recordings in monkey auditory cortex to confirm its presumed contribution to the later components of the F0_ENV_ response (Gnanateja et al., 2021). Additional work will need to focus on recordings in midbrain to confirm the contribution of these structures to FFR_ENV_ and FFR_TFS_.

It is known that the FFR_TFS_ can mirror the formant structure of the underlying vowel (Arenillas-Alcón et al., 2021). The current experiments were performed exclusively using the vowel /yi/, so it remains an open question if and how the F0_TFS_ responses may be modulated by the formant structure of different vowels.

The current linear models do not consider the longer timescale contextual effects that modulate the FFR_ENV_ and FFR_TFS_, which are attributed to putative corticofugal pathways. Future work needs to incorporate the potential role of stimulus context to improve explained variance and to comprehensively characterize the contribution of bottom-up and top-down pathways to the FFR_ENV_ and FFR_TFS_ (Chandrasekaran et al., 2009; Xie et al., 2018).

### Conclusion

Based on our studies in the rhesus macaque, we conclude that the deconvolution method can be used to compress complex and high-dimensional FFR_ENV_ and FFR_TFS_ to stimuli with time-varying pitch into a short and interpretable F0_ENV_ and F0_TFS_ response. The deconvolution method captures a decent amount of variance for the FFR_TFS_ and a substantially larger amount of the variance of the FFR_ENV_. The different latencies of the peaks and troughs likely reflect the sequential activation of structures along the auditory pathway, and may at some point be useful to map altered FFR_ENV_ and FFR_TFS_ in disease to altered function in specific brain regions.

There are already a large number of different ways to analyze FFR_ENV_ and FFR_TFS_, including broadband timing, F0 periodicity, phase consistency, and stimulus response correlation, to name just a few (Krizman & Kraus, 2019) that primarily reflect encoding fidelity. We propose that the value of the deconvolution approach arises from three main points: (1) the F0 responses are a lower-dimensional summary that captures and condenses much of the variance of the original FFR_ENV_ and to a lesser degree of the FFR_TFS_; (2) the latency of different features of the F0_ENV_ and F0_TFS_ responses is meaningful, and likely reflects the latency of different generators, thus linking altered F0_ENV_ and F0_TFS_ responses to specific anatomical substrates; and (3) the F0_ENV_ and F0_TFS_ responses can be measured with higher signal-to-noise ratio than the raw signals, thus providing an opportunity for increasing the sensitivity and power of subsequent statistical analyses.

## Frequency Following

Response (FFR): Umbrella term for several types of electrophysiological potentials that closely follow the periodicity of complex sounds such as speech.
Envelope FFR (FFR_ENV_): Reflects neural responses to periodic fluctuations of envelopes of various carrier frequencies at the rate of the fundamental frequency.
F0 response, or convolution kernel: The hypothetical neural response to the onset of an individual F0 cycle. “Convolution kernel” (or kernel for short) is a mathematical term that refers to the fact that the F0 response is a kernel estimated via deconvolution.
Temporal fine structure of the FFR, or spectral FFR (FFR_TFS_): Reflects the entrainment of neural responses to individual cycles of carrier frequencies below a certain physiological limit.
